# Virulence factors of *Moraxella catarrhalis* outer membrane vesicles are major targets for cross-reactive antibodies and have adapted during evolution

**DOI:** 10.1038/s41598-018-23029-7

**Published:** 2018-03-21

**Authors:** Daria Augustyniak, Rafał Seredyński, Siobhán McClean, Justyna Roszkowiak, Bartosz Roszniowski, Darren L. Smith, Zuzanna Drulis-Kawa, Paweł Mackiewicz

**Affiliations:** 1 Department of Pathogen Biology and Immunology, Institute of Genetics and Microbiology, University of Wroclaw, Przybyszewskiego 63/77, 51-148 Wroclaw, Poland; 2 Department of Physiology, Wroclaw Medical University, T. Chalubinskiego 10, 50-368 Wroclaw, Poland; 3 Department of Physical Chemistry of Microorganisms, Institute of Genetics and Microbiology, University of Wroclaw, Przybyszewskiego 63/77, 51-148 Wroclaw, Poland; 4 School of Biomolecular and Biomedical Sciences, UCD O’Brien Centre for Science West, B304 Dublin, Ireland; 5 Applied Sciences, University of Northumbria, Ellison Building EBD222, Newcastle upon Tyne, NE1 8ST UK; 6 Department of Genomics, Faculty of Biotechnology, University of Wrocław, Joliot-Curie 14a, 50-383 Wrocław, Poland

## Abstract

*Moraxella catarrhalis* is a common human respiratory tract pathogen. Its virulence factors associated with whole bacteria or outer membrane vesicles (OMVs) aid infection, colonization and may induce specific antibodies. To investigate pathogen-host interactions, we applied integrated bioinformatic and immunoproteomic (2D-electrophoresis, immunoblotting, LC-MS/MS) approaches. We showed that OMV proteins engaged exclusively in complement evasion and colonization strategies, but not those involved in iron transport and metabolism, are major targets for cross-reacting antibodies produced against phylogenetically divergent *M. catarrhalis* strains. The analysis of 31 complete genomes of *M. catarrhalis* and other *Moraxella* revealed that OMV protein-coding genes belong to 64 orthologous groups, five of which are restricted to *M. catarrhalis*. This species showed a two-fold increase in the number of OMV protein-coding genes relative to its ancestors and animal-pathogenic *Moraxella*. The appearance of specific OMV factors and the increase in OMV-associated virulence proteins during *M. catarrhalis* evolution is an interesting example of pathogen adaptation to optimize colonization. This precisely targeted cross-reactive immunity against *M. catarrhalis* may be an important strategy of host defences to counteract this phenomenon. We demonstrate that cross-reactivity is closely associated with the anti-virulent antibody repertoire which we have linked with adaptation of this pathogen to the host.

## Introduction

*Moraxella catarrhalis* is an important human-restricted pathogen responsible for sinusitis and otitis media in children as well as infections of the lower respiratory tract, causing exacerbation of chronic obstructive pulmonary disease in adults^1,2^. The most promising vaccine candidates against *M. catarrhalis* have been shown to induce protective immunity in pulmonary clearance of the bacteria in animal models^3,4^. In human studies, the high antibody levels against few vaccine candidates were correlated with reduced bacterial carriage^5^. Nevertheless, there is currently no licensed vaccine against *M. catarrhalis*^6^. This species is thought to be composed of two distinct lineages, namely the more pathogenic sero-resistant (SR) strains and the less pathogenic sero-sensitive (SS) ones^7,8^. These two groups of strains show separate evolutionary histories and contain a distinct subset of lineage-specific genes, 33 and 49, respectively^9^. Some of the genes unique to the SR lineage are involved in phosphate metabolism but others do not have assigned functions. However, most of the characterized virulence factors are present in the core genome of both SR and SS strains. The difference in pathogenicity correlates with the clinical observation that most strains isolated from diseased patients possess the sero-resistant features^10^. The sero-resistant strains isolated from different patients are very similar with respect to their virulence, metabolic potential, CRISPR-cas, mobile genetic element content, gene content and chromosomal synteny^11^. The fact that nearly three-quarters of the genes are common, including those associated with virulence, indicates that a range of isolates of *M. catarrhalis* may be controlled by vaccination. A potential distinction between the isolates could result from an extremely small number of genes or epigenetic phenomena^11^. Such epigenetic regulation of multiple gene expression via the phase-variable DNA methyltransferase (ModM) regulon was recently reported for *M. catarrhalis* phase varions associated with otitis media^12^.

The mechanisms of colonization and pathogenesis of *M. catarrhalis* have been extensively studied and many virulence factors have been identified to date. The most important virulence strategies involve: (1) evasion of complement-mediated killing mainly via interference with regulatory proteins^13–15^; (2) polyclonal, non-specific B cell activation and redirecting of adaptive immunity^16^; (3) hiding inside lymphoid tissue, which is the main reservoir facilitating the host invasion^17^; (4) formation of biofilm^18,19^; and (5) participation in protease-antiprotease imbalance^20^. Some of these strategies can be driven in part by the release of outer membrane vesicles (OMVs), which contain several virulence factors facilitating the delivery of periplasmic and outer membrane components to the host^14,21^. Moreover, OMVs can favour pathogen coexistence and colonization after their interaction with the other bacterial species^22,23^.

Many immune-relevant and shared microbial epitopes stimulate the production of intraspecies and interspecies cross-reacting antibodies with high frequency and have cross-protective efficiency^24,25^.

The major arm of defence against *M. catarrhalis* relies on antibody-dependent mechanisms^6^. We have previously reported that some of them, including bactericidal, opsonophagocytic and adhesion blocking protective function, can be driven *in vitro* by cross-reactive antibodies that primarily recognize outer membrane proteins (OMPs)^26^. To confer an anti-virulent strategy, the antibodies directed against *M. catarrhalis* may also act in concert with unique humoral mediators such as neuropeptides^27^. To investigate the nature and basis of our previous observations and define the specific profile of OMPs targeted by cross-reactive antibodies, we performed an immunoproteomic analysis of OMVs from two clinical strains of *M. catarrhalis* (Mc6 and Mc8) which differ in phenotypic properties, lipooligosaccharide (LOS) type, source of origin^26^ and belong to distant phylogenetic lineages. Although a comprehensive proteomic analysis of OMVs from one *M. catarrhalis* RH4 strain was recently published^28^, immunoproteomic studies have not been performed to date. The immunoproteomic analysis of OMVs was characterized by 2D-electrophoresis associated with LC/MS mass spectrometry and carried out by cross-immunoreactivity experiments. Moreover, we performed extensive bioinformatics analyses of OMV proteins including four newly sequenced and all previously published *Moraxella* complete genomes to study features of these proteins from a genomic and evolutionary perspective with particular emphasis on their role in virulence.

## Results

### Phylogenetic relationship between the studied genomes

**Figure 1:**
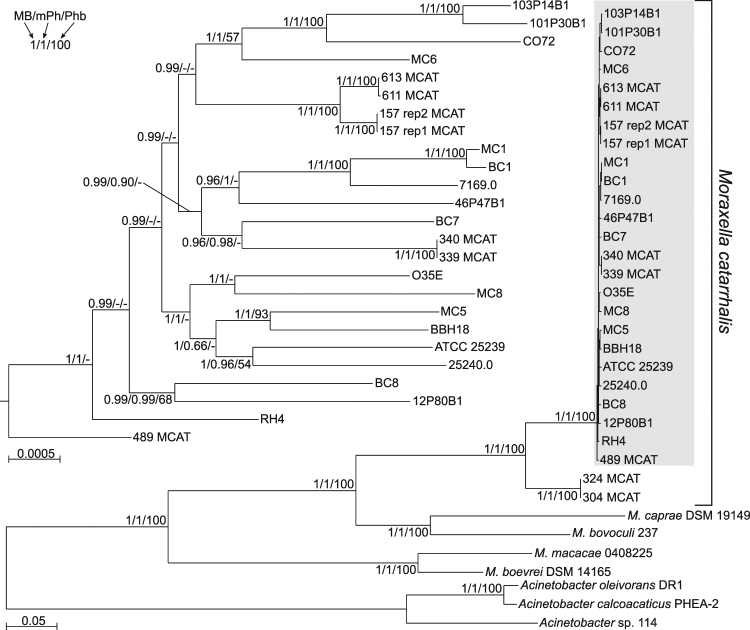
Phylogenetic relationships between *Moraxella* and *Acinetobacter* genomes. MrBayes tree based on the concatenated alignments of sequences from 739 orthologous groups represented 31 *Moraxella* genomes and three members of *Acinetobacter* genus. The part of tree marked by the grey rectangle is shown on the left in the larger scale. Numbers at nodes, in the order shown, correspond to: posterior probabilities estimated in MrBayes (MB), support values obtained by the approximate likelihood ratio test based on a Shimodaira-Hasegawa-like procedure (Anisimova and Gascuel O. 2006) calculated in morePhyML (mPh) and bootstrap values obtained in PhyML (Phb). Values of the posterior probabilities, SH-like branch supports and bootstrap percentages lower than 0.50 and 50%, respectively, were omitted or indicated by a dash “−”.

To determine the evolutionary relationships of the newly sequenced genomes (Mc1, Mc5, Mc6, Mc8) in comparison to other *M*. *catarrhalis* strains **(**Supplementary Table S1**)**, we performed phylogenetic analyses of 34 complete genomes using alignments of 739 orthologous groups of proteins **(**Fig. 1**)**. Trees obtained by different methods show very similar topologies to trees based on the presence and absence of the orthologous groups **(**Supplementary Fig. S1**)**. All *M*. *catarrhalis* strains create a fully supported monophyletic clade and exclude the four other *Moraxella* species isolated from animals. Apart from two *M*. *catarrhalis* strains, 324 MCAT and 304 MCAT, the other strains are very tightly grouped and are characterized by minimal divergence in comparison to other studied bacteria. The newly obtained strains, are distributed across various branches of the *M*. *catarrhalis* phylogenetic tree. Two *M. catarrhalis* strains, Mc6 and Mc8, selected for proteomic analysis, are placed in different clades separated by many other lineages, therefore representing relatively distant phylogenetic lineages considering intraspecies variations. Furthermore, both strains are phenotypically different in LOS type, autoagglutination ability, adherence potency, biofilm formation and source of origin as described previously^26^.

### Profiles of immunoreactive OMV proteins from *M. catarrhalis* strains

The OMV protein profiles from the strains Mc6 and Mc8 were compared by 2-D electrophoresis (2D-SDS PAGE). The resulting peptide mass fingerprints were analysed using a *M. catarrhalis* protein data set including 18 proteins. Only minor variations in the patterns of resolved protein spots between the strains Mc6 and Mc8 were obtained with respect to relative concentration, charge and molecular weight of a few proteins **(**Table 1**)**. Our proteomic studies identified ten additional OMV proteins to those already described by Schaar *et al*. (2011), which display molecular masses between 15 and 120 kDa **(**Supplementary Data S1**)**. They represent different functional groups. Four of them are probably involved in binding and transport of iron ions: FbpA, MhuA, LbpB and TbpA. Others are glutamate dehydrogenase (GdhA), ubiquitous surface protein UspA1, structural protein (MipA), polyisoprenoid-binding periplasmic protein (YceI), molecular chaperone (GroEL) and dihydrolipoamide dehydrogenase (Lpd). With the exception of TbpA, identified in Mc6 OMVs (LC-MS/MS: MW 119.4, accession number SQ01_05725, sequence coverage 34%, score 1734), nine of them were immunogenic **(**Table 1**)**.

**Table 1:** Immunogenic proteins determined by LC-MS/MS of excised spots found by immunoblotting on 2DE gels.

Protein description and name	Functional category^1^	MW (kDa)^2^	Strain	Accession number	Sequence coverage (%)	Score^3^
Outer membrane protein CD (OMP CD)	Cell envelope biogenesis, outer membrane^1^, cell binding, serum resistance	48.3	Mc6	SQ01_01315	23–56	328–1213
			Mc8	SQ02_01320	25–44	464–1064
Outer membrane protein E (OMP E)	Lipid metabolism^1^, fatty acid transport, serum resistance	49.3	Mc6	SQ01_04940	69–71	1473–1720
			Mc8	SQ02_04980	45–67	1105–1995
Outer membrane protein (CopB), TonB-dependent siderophore receptor	Inorganic ion transport and metabolism^1^, serum resistance	82.8	Mc6	SQ01_06490	30–58	1115–2204
			Mc8	SQ02_06550	33–50	1260–2019
Ubiquitous surface protein A (UspA1)	Cell division and chromosome partitioning^1^, cell binding, serum resistance	93.3	Mc6	SQ01_03435	24–41	596–1011
			Mc8	SQ02_03470	22–23	787–782
Outer membrane porin (OMP M35)	Cell envelope biogenesis, outer membrane^1^, transport and binding proteins	38.0	Mc6	SQ01_03260	38	533
			Mc8	SQ02_03275	78	1286
Transferrin binding protein B (TbpB)	transport and binding proteins	76.2	Mc6	SQ01_05710	22–38	884–1140
			Mc8	SQ02_05755	21	710
Lactoferrin binding protein (LbpB)	Transport and binding proteins	100.1	Mc6	SQ01_07800	10	353
			Mc8	SQ02_07885	13	558
Haemoglobin-utilization protein, TonB-dependent receptor, (MhuA)	Inorganic ion transport and metabolism^1^, transport and binding proteins	107.6	Mc6	SQ01_05485	37–40	1422–1460
			Mc8	SQ02_05535	ND	ND
Structural protein (MipA)	Cell envelope biogenesis, outer membrane^1^	28.6	Mc6	SQ01_04155	53–60	498–577
			Mc8	SQ02_04180	75	799
Fe(3+) ABC transporter substrate-binding protein (FbpA)	Inorganic ion transport and metabolism^1^	35.7	Mc6	SQ01_07425	ND	ND
			Mc8	SQ02_07515	58–73	849–1080
Molecular chaperone (GroEL)	Posttranslational modification, protein turnover, chaperones^1^	57.4	Mc6	SQ01_02125	ND	ND
			Mc8	SQ02_02135	60	1721
Dihydrolipoamide dehydrogenase (Lpd)	Energy production and conversion^1^	51.1	Mc6	SQ01_00050	29	737
			Mc8	SQ02_00050	46	1204
Hypothetical protein with TPR domain (HP Mc4)	General function prediction only^1^	30.1	Mc6	SQ01_03330	46	554
			Mc8	SQ02_03345	62	758
Outer membrane beta-barrel protein OMPJ)	Cell envelope biogenesis, outer membrane^1^	18.9	Mc6	SQ01_07075	ND	ND
			Mc8	SQ02_07135	34	246
Polyisoprenoid-binding periplasmic protein (YceI)	General function prediction only^1^	21.2	Mc6	SQ01_05855	ND	ND
			Mc8	SQ02_05915	63	528
Membrane protein (OMP G1b)	Cell envelope	26.9	Mc6	SQ01_02650	29	448
			Mc8	SQ02_02660	ND	ND
Membrane protein, Opa-like protein A (OlpA)	Cell envelope biogenesis, outer membrane^1^	23.3	Mc6	SQ01_01010	50	296
			Mc8	SQ02_01010	ND	ND

^1^Functional category according to search results of Cluster of Orthologous Groups (COGs); other descriptions were taken from various references;

^2^Theoretical molecular mass was determined by Mascot;

^3^Score ranges represent MS/MS ion scores determined by peptide mass fingerprinting. Only scores deemed to be significant by Mascot (p < 0.05) were presented.

ND - not done as there was no reactivity observed on blots.

A pseudogene SQ02_07885 showing similarity to the gene encoding lactoferrin binding protein (LbpB) was annotated in the Mc8 genome. Therefore, it has been later rechecked and verified by resequencing of 1.2 kb PCR product. The sequence of this truncated gene contains many stop codons and indels which disrupt its potential amino acid sequence and cause frame shifts. The first stop codon appears after 2214 nucleotides giving the potential amino acid product with the length of up to 738 residues. The original amino acid sequence would result in 906 residues. However, the truncated sequence contains a complete protein domain (pfam01298, Lipoprotein_5, Transferrin binding protein-like solute binding protein) present in its full homologues. We confirmed that the shortened protein retains its immunogenic properties. This protein product was identified in immunoblots and showed reactions with antisera as shown below.

### Immunoblot analysis of OMV proteins reveals a specific pattern of cross-reactivity with antibodies

To study immunoreactive proteins present in OMVs from Mc6 and Mc8 strains, one homologous anti-serum and two cross-reactive, serotype-different murine sera were selected. These sera, namely anti-Mc1, anti-Mc6 and anti-Mc8 which were raised in response to whole bacteria in order to better mimic the natural reaction of the host, showed significant cross-protective bactericidal and anti-adhesive activity *in vitro*^26^. The characteristics of used antisera is shown in Table 2. The antisera were specific against strains representing separate lineages in the phylogenetic tree of *M*. *catarrhalis* strains **(**Fig. 1**)** and consequently represent a wide phenotypic variation within this species. The advantage of studying *Moraxella* cross-reactivity using mouse sera is connected with the fact that all antibodies specific against *M. catarrhalis* antigens in mouse are produced *de novo* without any interference from pre-existing antibodies.

**Table 2:** Characteristics of murine antisera.

Serum	Strain Mc6			Strain Mc8		
	Titer^1^	Avidity index^2^	Bactericidal titer^3^	Titer^1^	Avidity index^1^	Bactericidal titer^3^
anti-Mc1	246,254	46 ± 4.2	261	255,242	34 ± 0.7	75
anti-Mc6	2,140,484	79 ± 1.4	607	751,395	53 ± 1.4	317
anti-Mc8	255,290	57 ± 0.7	234	1,001,067	43 ± 2.8	720
pre-immune	<500	ND	<3	<500	ND	<3

^1^The end-point of IgG ELISA titer against OMVs is defined as reciprocal value of the antiserum dilution giving an absorbance of control non-immunized mouse serum enlarged by two standard deviations (2 SD). The values are presented as geometric mean titers from two independent experiments.

^2^Avidity was tested by OMVs ELISA elution assay, using a chaotropic agent 1.5 M sodium thiocyanate (NaSCN) to disrupt weak antigen-antibody binding. The results are expressed as mean avidity index (AI) with SEM where AI = (OD_490_ with NaSCN/OD_490_ without NaSCN) × 100.

^3^The titer was defined as the reciprocal value of the highest dilution of antiserum at which ≥50% killing of the target strain was observed. The results were published in our previous paper^26^.

In immunoproteomic studies, a combination of 2D-gel electrophoresis, western blot analysis and LC-MS/MS was performed. The spots that were immunoreactive with the cross-reactive and homologous antibodies were matched to the stained reference gel by comparison of their electrophoretic position, size and shape. The most immunoreactive proteins spanned a broad range of pI values between 4.5 and 10 and molecular mass from 20 to 80 kDa. Overall, 24 Mc6 and 27 Mc8 immunoreactive spots were successfully identified by mass spectrometry (out of 51 Mc6 and 52 Mc8 immunoreactive spots, respectively), representing 13 and 14 different proteins **(**Table 1**)**. The number of immunoreactive spots in Mc6 and Mc8 was similar when probed with homologous antisera, up to 25% lower for more-potent heterologous antisera (anti-Mc6 or anti-Mc8) and up to 70% lower for less-potent heterologous antisera (anti-Mc1).

**Figure 2:**
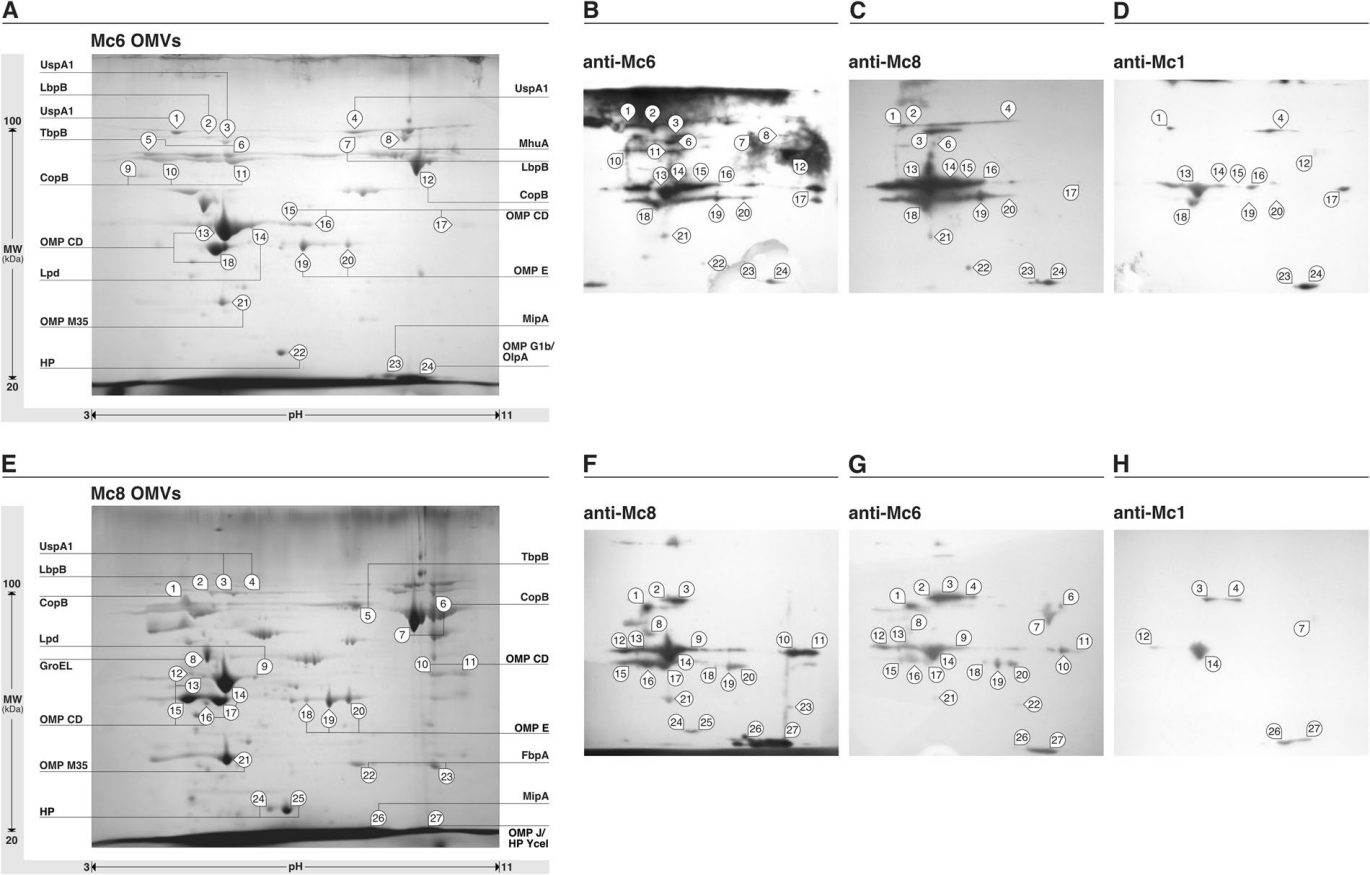
Immunoproteomic analyses of *M. catarrhalis* OMV preparations. After 2D-gel electrophoresis OMV proteins were visualized by Coomassie staining (**A**,**E**). 2D immunoblots probed with homologous antisera (**B**,**F**), stronger cross-reactive antisera (**C**,**G**) and weaker cross-reactive antisera (**D**,**H**). Molecular size markers are indicated on the left. The exposure times for chemiluminescent substrate were exactly the same for each presented blot. Gel and blot images are cropped and brightness/contrast-adjusted for better readability. Original images are shown in Supplementary Fig. S2.

**Figure 3:**
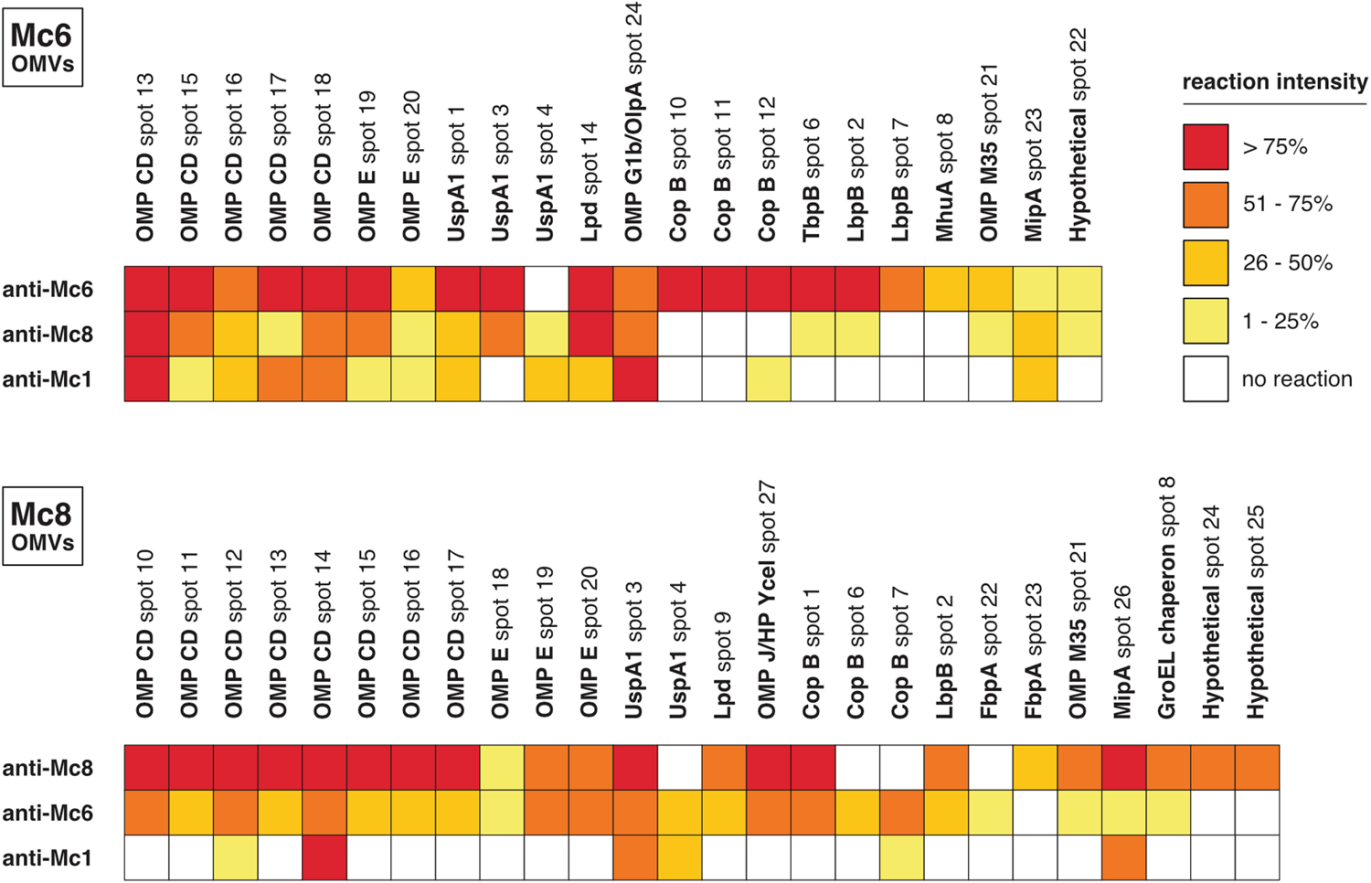
Heatmap of immunoreactive spot intensities. Reactivity of murine post-immunization sera with OMVs proteins was estimated semi-quantitatively on a quartile scale. Only proteins that reacted with at least one antisera are shown.

The analyses allowed the identification of a set of common proteins with different functions, mostly classified as outer membrane proteins, which were recognized by all three antisera used. Several of these immunoreactive proteins, including OMP CD were identified from more than one spot, often with the same molecular weight but with different pI values. These are likely to represent isoforms of the same protein that retain their immunogenicity. The analyses allowed also for an unambiguous identification of several proteins which showed qualitative and quantitative differences in immunoreactivity. As presented in Figs 2 and 3, the most immunoreactive -cross-reacting proteins showing the highest staining intensities for IgG antibodies seem to be the outer membrane proteins involved in bacterial adhesion (OMP CD, UspA1) or complement resistance (UspA1, OMP E, OMP CD, OMP J/OlpA/OMP G1b). On the other hand, the predicted proteins involved in iron acquisition such as TbpB or LbpB, including the truncated version of LbpB in Mc8, were rather weakly or not recognized by cross-reactive antibodies, while their recognition by homologous sera was abundant for the relevant studied strain. A similar pattern of reactivity was observed for protein engaged in iron acquisition by hemoglobin utilization such as MhuA. Iron-binding protein CopB gave a variant and strain-dependent pattern of cross-reactivity. The lack of cross-reactivity was noticed for periplasmic ferric binding protein FbpA abundantly expressed in Mc8. There were also a few proteins predicted to be both cytoplasmic and surface exposed. Among them were Lpd and GroEL chaperone which showed moderate immunoreactivity in both strains while probing with anti-Mc6 or anti-Mc8 sera in heterologous manner. Taken together, the more-potent cross-reactive antisera (anti-Mc6 or anti-Mc8) recognized 11 different proteins with high or moderate spot intensities, whereas the less potent antiserum (anti-Mc1) recognized between 5 and 7 different proteins (depending on the strain used) thus showed lower intensities of reactions in general. The original full-length images of 2D-gels and blots can be seen in Supplementary Fig. S2.

Due to the size limitations of 2D electrophoresis for the certain high molecular weight (HMW) proteins of *M. catarrhalis* such as Hag/MID or UspA2, their presence in OMVs and their reactivity with antisera were performed by classical SDS-PAGE and Western blot. Strong cross-reactivity was only observed for the ~250 kDa HMW antigen (presumably Hag/MID) of Mc6 and Mc8. There was no cross-reactivity for the other HMW protein, which was abundantly visible on the gel, with a MW in excess of 300 kDa. OMVs from Mc6 but not Mc8 *Moraxella* strains heated at 37 °C and 100 °C for 10 min showed the presence of this HMW antigen, which indicates the predicted presence of UspA2 antigen with very high molecular size and a multimeric structure that was not reduced by heating at 100 °C^29^. The results for HMW antigens are presented in Supplementary Fig. S3.

### Potential epitopes in OMV proteins subjected to cross-reaction studies

It is interesting to hypothesize on the reasons why some proteins were able to react with antisera elicited by different *Moraxella* serotypes while others were not. One could assume that it is related to various level of sequence conservatism of these proteins. Proteins with lower sequence divergence should respond to the cross-reactions with greater probability and intensity than more divergent ones. Indeed, the homologous proteins involved in strong positive cross-reactions were characterized on average by a significantly smaller mean percentage difference between their entire sequences (2.85%) than the proteins engaged in weak or no cross-reactions (8.04%), (p-value = 0.04). However, the global sequence divergence may not well reflect the variation/conservatism of epitopes responsible for the cross-reactivity.

Therefore, we searched the sequences of proteins for potential epitopes using 15 bioinformatic prediction methods. As potential epitopes, we selected sites that were recognized by more than half of the approaches. Plots showing the prediction of epitope sites along sequences of the studied proteins are presented in the Supplementary Fig. S4. Supplementary Data S2 includes the list of individual amino acid residues that were predicted to be involved in the epitopes of individual proteins. The fractions of the sites predicted as epitopes were quite variable (Supplementary Data S3**)**. Assuming the 50% prediction threshold, the percent of these sites in sequences range from 0.4% (Lpd protein from Mc1 and Mc6 strains) to 22.1% (LbpB protein from Mc8 strain). Proteins that were not involved in strong cross-reactions had a significantly larger contribution of predicted epitope sites in their sequences (on average 9.9 ± 6.2% SD), than the proteins engaged in such cross-reactions (6.1 ± 3.8% SD, t-test, p-value = 0.012). The predicted epitope sites of homologous proteins engaged poorly or not at all in strong cross-reactions were also characterized on average as having almost twice the percentage differences between their sequences (12.9%) as the epitope sites of homologous proteins involved in the cross-reactions (6.8%).

### Distribution of OMV proteins in the studied genomes

To study and characterize OMV proteins in a more general functional and evolutionary context, we first clustered all protein sequences from four newly sequenced and 23 publicly available full genomes of *M. catarrhalis* as well as four other *Moraxella* species into orthologous groups (Supplementary Table S2**)**. Three representatives of *Acinetobacter* (family *Moraxellaceae*) were added as reference genomes because it is the most closely related genus (with the published complete genome) to *Moraxella*. The analyses revealed 4780 orthologous groups represented by at least two of the 34 studied genomes and 779 groups had at least one protein in each of the genomes **(**Supplementary Data S4**)**.

**Figure 4:**
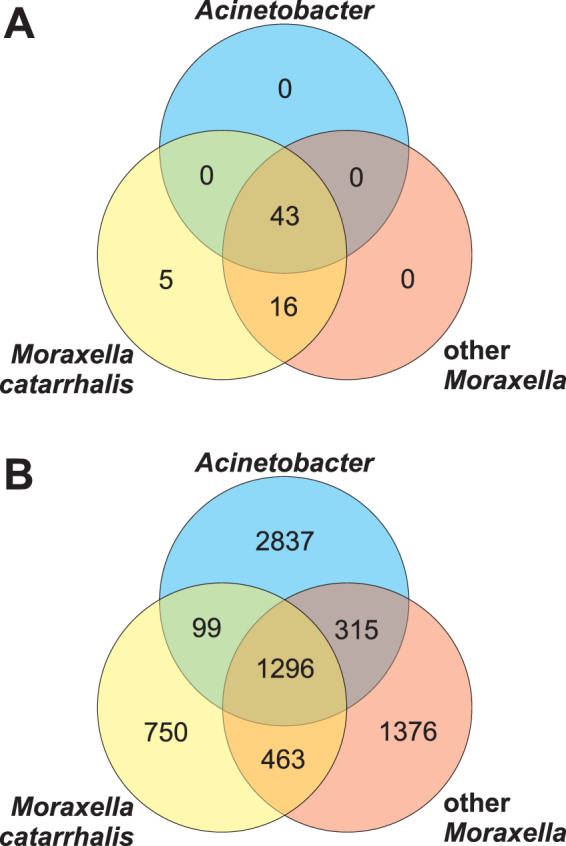
Distribution of orthologous group for OMV proteins (**A**) and total proteins (**B**) found with OrthoMCL across three sets of genomes: *Acinetobacter*, *Moraxella catarrhalis* and other *Moraxella* species. The group was included if it was represented by at least one genome in the set. The unique proteins not classified into groups were also included.

OMV proteins were assumed to be those that were previously identified in proteomic studies by Schaar *et al*. (2011) and by us. Using homology searches, we assigned the OMV proteins to the orthologous groups. These proteins were recognized in 64 orthologous groups **(**Supplementary Data S1**)**, which contained 1995 annotated proteins in total from the studied genomes. Among the 64 OMV protein groups, 43 were conserved and present in at least one representative of *Acinetobacter* as well as in *M. catarrhalis* and other *Moraxella* species **(**Fig. 4A**)**, while 24 were found in all genomes studied. However, a substantial proportion (25%), i.e. 16 of the OMV protein groups were represented only in *Moraxella* and five OMV protein groups were restricted to *M. catarrhalis*: IcIR (W2387), Lgt1 (W2401), OppB (W2484), cHP Mc10 (W2552) and HP Mc12 (W2666) **(**Supplementary Data S1**)**. Interestingly, no such unique OMV proteins were found in *Acinetobacter* or in other *Moraxella* species although their genomes encode many unique proteins of other types **(**Fig. 4B**)**. A likelihood ratio test (G-test) of independence showed significant differences between the distribution of orthologous group for total and OMV proteins (p-value < 2.2E-16).

Forty four OMV protein groups were found in all 27 *M. catarrhalis* strains and 32 were identified in all 31 *Moraxella* strains examined. Homologues to hypothetical protein MCR_0740 were the least represented in the genomes because they were found in only 10 *M. catarrhalis* strains. Genes coding for a phage-related protein PmpL and ubiquitous surface protein A UspA1 were subjected to duplication in several *M. catarrhalis* strains.

### Functional categories of OMV proteins

The majority of all identified OMV proteins were grouped into three functional categories: (1) Cell wall/membrane/envelope biogenesis, (2) Translation, ribosomal structure and biogenesis and (3) Inorganic ion transport and metabolism. Each of these categories contained more than 10% OMV proteins. In turn, the functional groups most enriched in the OMV proteins in comparison to non-OMV proteins were: Secondary metabolites biosynthesis, transport and catabolism; Cell wall/membrane/envelope biogenesis; Cell cycle control, cell division, chromosome partitioning/Cytoskeleton and Extracellular structures. They were represented twice as much among OMV proteins than non-OMV proteins **(**Supplementary Table S3**)**. It is also interesting to compare distribution of total proteins into functional categories across *M. catarrhalis* strains infecting human upper respiratory tract and *Moraxella* isolated from animals **(**Supplementary Table S4**)**. For many categories, the differences between the fractions were statistically significant. The human pathogens had over twice as many proteins involved in: Amino acid transport and metabolism; Defence mechanisms; Nucleotide transport and metabolism and Coenzyme transport and metabolism.

### Changes in content of genes for OMV proteins in the studied genomes

**Figure 5:**
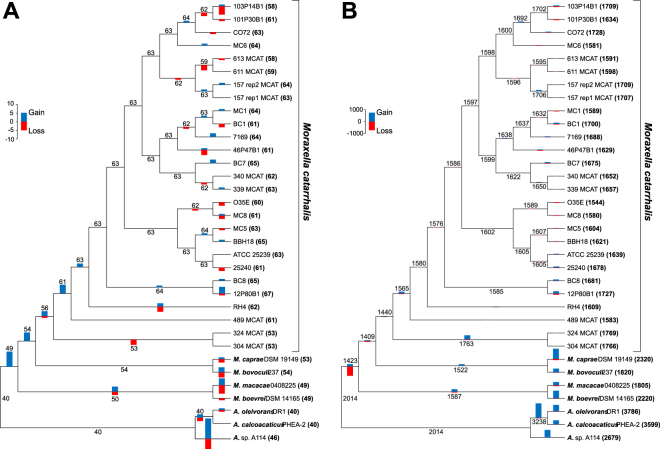
Gains and losses of genes for OMV proteins (**A**) and all annotated protein sequences (**B**) during evolution of *Moraxella* and *Acinetobacter* genomes. The number of gene gains and losses per phylogenetic lineage were presented by bars. Ancestral gene contents in genomes were shown by numbers at tree branches and the current gene contents were presented by numbers in parenthesis at individual genomes.

Using the orthologous group distribution and the phylogenetic tree of the studied genomes, we estimated sizes of gene content in ancestral genomes as well as gains and losses of genes in individual phylogenetic lineages. We compared the results of these studies between genes encoding OMV proteins and all other annotated protein sequences **(**Fig. 5**)**.

The size of the ancestral OMV protein set for the common ancestor of *Moraxella* and *Acinetobacter* was assessed to be 40 **(**Fig. 5A**)**. This number remained almost unchanged during the evolution of *Acinetobacter* genomes because they encode currently 40 or 46 such proteins. In contrast, during the evolution of *Moraxella*, the number of genes encoding these proteins continuously increased. Its successively diverging lineages comprised an increasing number of these genes: 50 (the common ancestor of *M. boevrei* and *macacae*), 54 (the common ancestor of *M. bovoculi* and *caprae*), 56 (the common ancestor of all *M*. *catarrhalis* strains), 61 (the common ancestor of *M*. *catarrhalis* strains excluding the basal 324 MCAT and 304 MCAT strains). The subsequent increase in this gene content resulted in 58 to 67 such genes per genome in *M*. *catarrhalis* strains that evolved after separation of the 324 MCAT and 304 MCAT strains. Among the evolved genomes, only small fluctuations in the gene content has occurred: up to 5 losses (the lineage of 103P14B1) and up to 4 gains (the lineage of 1280B1 strain).

The increase in the number of genes coding for OMV proteins does not seem to be an artefact related to the homology searches in the closely related genomes of *M*. *catarrhalis* versus the others because the same analysis performed with all protein-coding genes did not reveal the trend typical of the genes for OMV proteins. Furthermore, the whole proteome showed the opposite trend **(**Fig. 5B**)**. In contrast to the content of genes encoding OMV proteins, ancestors of *Moraxella* decreased their global protein-coding gene content by almost 600 genes, whereas *Acinetobacter* genomes increased their genomes by 665 to 1772 genes in comparison to their common ancestor with 2014 genes. Some increase in the global gene content in *Moraxella* arose much later during evolution and rather independently in individual lineages. The most gene-abundant genomes evolved in *Moraxella* isolated from animals (1805 to 2320 genes), while human pathogenic *M*. *catarrhalis* strains encode only 1544 to 1769 proteins.

**Figure 6:**
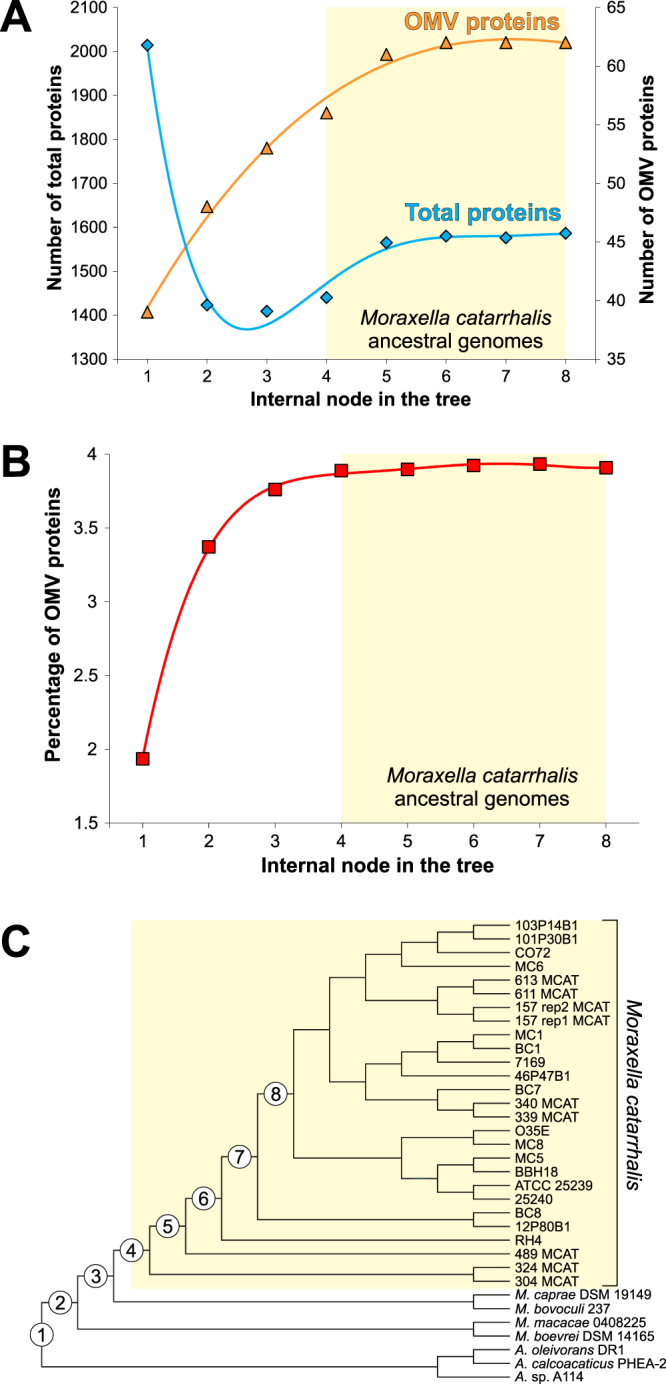
Changes in gene content during evolution of *Moraxella* and *Acinetobacter* genomes. Number of genes for total proteins and OMV proteins estimated for ancestral genomes (**A**) in subsequent internal nodes from 1 to 8 in the phylogenetic tree presented in (**C**). Percentage of OMV proteins coded in the ancestral genomes from subsequent internal nodes (**B**).

These trends in gene content alteration are apparent in Fig. 6A, where the numbers of genes for total or OMV proteins estimated for ancestral genomes were plotted along the subsequent internal nodes of the phylogenetic tree **(**Fig. 6C**)**. The number of OMV protein genes clearly increased during evolution and reached a stable maximum for *M. catarrhalis* ancestral genomes. Simultaneously, the total number of protein-coding genes rapidly dropped and subsequently increased only slightly. As a result of these tendencies, the percentage of OMV proteins coded in the ancestral genomes doubled, from about 2% to almost 4% **(**Fig. 6B**)**.

Since OMV proteins are common antigens, it was interesting to establish if their sequences showed a greater variation than other types of proteins. The median percentage difference of the OMV proteins was slightly larger than that of the other proteins: 16.2% and 15.4%, respectively.

## Discussion

Proteins associated with OMVs are engaged in diverse biological processes enabling infection and colonization of host by pathogens. Many of them are important virulence factors enhancing the potential to cause diseases^30^. Our comprehensive computational analyses of 31 complete *Moraxella* genomes and three representatives of *Acinetobacter* showed that OMV proteins identified in *M*. *catarrhalis* are organized in 64 orthologous groups and involved in various functions, especially those related to cell membrane and inorganic ion transport. Interestingly, we found that *M*. *catarrhalis* genomes are significantly enriched in these OMV proteins in comparison to other *Moraxella* species isolated from animals. It indicates that these proteins are not conserved across other taxa and have been quite recently acquired by *M*. *catarrhalis*. The acquisition of their genes can be associated with the emergence of these human-specific pathogens and is confirmed by the leading role of OMV in the increasing infectivity, pathogenicity and virulence of these bacteria. It has been documented that proteins incorporated in *M. catarrhalis* OMVs can play a role in complement evasion, epithelial adhesion, redirection of adaptive humoral response, beta-lactam antibiotic cleavage and biofilm formation^16,23,28,31^. On the other hand, the lack of OMV enrichment in *Moraxella* species infecting other mammals could suggest either a major involvement of other virulence factors (e.g. cytotoxins or type IV pili in *M. bovis and M. bovoculi*) or alternatively their lower pathogenicity^32,33^.

Since OMV components in general are strong immunomodulatory antigens, they can significantly influence the mammalian immune system^21,34^ and stimulate it to develop effective cross-protective defence^35,36^ which in turn may influence the outcome of natural infections^37,38^. Recently, we have reported the wide range of effector anti-virulent functions fulfilled by cross-reacting antibodies against phenotypically different *M. catarrhalis* clinical strains^26^. In contrast to the previous proteomic studies on OMVs from RH4 stain of *M. catarrhalis*^28^, our present approach, focused on immunoproteomic analyses, revealed a particular set of antigens associated with virulence and cross-immunity that showed a similar pattern of responsiveness. The antigens that were highly cross-recognized by sera including OMPCD, UspA1, OMPE, OlpA/OmpJ and presumably Hag/MID, were specific proteins responsible for serum resistance/complement evasion and/or adhesion enhancement. Therefore, the basis of antibody-mediated action directed against heterologous *M. catarrhalis* strains seems to be multidirectional blocking of these pivotal virulence factors. The observed significant response of cross-reacting antibodies to cytosolic/surface exposed Lpd and in part also to GroEL chaperone seems to be in line with this, as it reveals quite potent immunogenic properties of proteins whose virulent potential is well described. To our knowledge, this is the first study showing the immunogenicity of Lpd. In addition, the involvement of *Pseudomonas* Lpd in inhibition of complement cascade has been demonstrated^39^. GroEL immunoreactivity was observed in *Burkholderia*^40^ infections. Likewise, the immunogenicity and protective efficacy of GroEL was confirmed for *Streptococcus pneumoniae* against a lethal infection in mice^41^. Although the surface exposition of GroEL and Lpd have not been directly confirmed to date for *M. catarrhalis*, their presence on the surface of other bacteria including *Neisseria meningitides*^42^ and *Pseudomonas aeruginosa*^43^ suggests a similar location. We were not able to detect UspA2 protein involved in serum resistance on blots, despite the confirmed presence of a heat-resistant HMW protein (size exceeds 300 kDa on the gel) at least for Mc6. Its identify is implied due to it being highly resistant to boiling, a biochemical feature of UspA2. The lack of immunoreactivity for UspA2 may be a result of phase variation^29^ or poor transfer to blotting membranes as in case of other bacterial HMW proteins^44^.

The apparent presence of specific cross-reactive patterns (the qualitative cross-reactive threshold) was next confirmed for a distinct group of abundantly expressed OMV antigens, involved in iron acquisition from Fe-sequestering host proteins. This group contained protein antigens which did not appear to be classified as pivotal virulence factors and were not recognized, or only weakly recognized, by cross-reacting antibodies. Interestingly, three of them, namely transferrin-binding protein (TbpB) and lactoferrin-binding protein (LbpB), as well as CopB (responsible for binding of both transferrin and lactoferrin) which gave strong homologous reaction in our study, have been reported to induce an antibody response in convalescent sera or sputum^1,45^. It supports their specific rather than cross-reactive recognition. We found no reactivity with TbpA and LbpA proteins encoded by two *Moraxella* strains. There is still no conclusive evidence for antibody against *M. catarrhalis* TbpA or LbpA in convalescent phase sera^45^, although such results exist for experimental meningococcal infection^46^. The inefficiency in TbpA refolding during the electroblotting procedure also cannot be ruled out. For others such as hemoglobin-binding protein (MhuA)^47^ or FbpA, responsible for iron transport from periplasmic space into the cell interior^48^, there is no data on their immunogenic significance to our knowledge. Although, the induction of functional neisserial anti-FbpA antibodies in an animal model has been described^49^, their bactericidal importance has not been confirmed^50^.

We found that epitope sequences of homologous non-virulent OMV proteins showing either weak or no cross-reactivity were more variable than those of virulent proteins involved in the strong cross-reactions. It clarifies why antibodies prepared on one strain cross-reacted with the other strain, most probably with the conserved epitopes. Therefore the quantitative increase in OMV protein content during evolution observed in this study could compensate the lack of any qualitative changes expressed by conservatism at the sequence level of many protein epitopes. Lack, or presence, of cross-reactivity may be due to amino acid replacements or deletions/insertions in the sequences. For example, we found sites in the sequence of TbpB proteins that are well recognized as epitopes in Mc6 strain by 11 and 12 methods but in strains Mc1 and Mc8 by much smaller number of the methods **(**Supplementary Fig. S5**)**. The difference in the prediction is associated with substantial changes in their sequences. Consistent with this, the proteins from the Mc6 strain showed practically no cross-reactivity with antisera prepared from the Mc1 or Mc8 strains. However, mutations cannot explain the complete variation in cross-reactivity, because a few proteins that have identical sequences were not recognized by the cross-reactive antibodies, e.g. FbpA from Mc1 and Mc8 strains, GroEL from Mc1 and Mc6 strains and OMP M35 from all three strains. This observation implies than other factors can influence the cross-reactivity, e.g. posttranslational modifications, different exposition of protein in OMVs, intrinsic features of antigenic epitope such as its accessibility and density or at least insufficient levels of certain cross-reactive antibodies^51–53^.

Taken together, the majority of cross-reactive antibodies was directed against several *M. catarrhalis* targets that are involved in serum resistance and/or promotion of bacterial attachment and as such contribute to the pathogenicity of bacteria. It is also difficult to judge their individual importance *in vivo*. The advantage for the host in having antibodies with a range of specificities against bacterial antigens associated with serum resistance is to facilitate an effective defence even if particular sets of (monovalent) antibodies are not present in the antiserum.

In conclusion, the cross-reactive humoral immune response elicited by the whole bacteria is directed against *M. catarrhalis* antigens engaged exclusively in complement evasion and colonization/invasion strategies but not those which participate in nutrient or compound acquisition. Despite the fact that strain-specific immune responses are a hallmark of many bacterial infections including those caused by *M. catarrhalis*^54^, the immunity based on cross-reacting anti-virulent activity of antibodies^26^ is likely to be an additional source of defence, which increases the likelihood of successful eradication of this human pathogen. The significant pattern of cross-reactivity against pivotal virulence targets is therefore advantageous for the host. On the other hand, cross-reactive immunity may have also significant evolutionary implications for bacteria virulence strategies. The observed twofold rise in the number of specific OMV proteins during evolution of *M. catarrhalis* is an interesting example of pathogen adaptation in successful colonization of the host and increasing virulence of this species. Our findings suggest that virulence factors associated with OMVs and cross-reactive antibodies are pivotal components of *M. catarrhalis-*host interaction and may have implications in the host-pathogen interplay. Further *in vivo* studies are needed to confirm the role of *M. catarrhalis* OMVs in cross-protection.

### Limitations of the data

The conclusions of our study are based on results performed with murine antisera developed to antigens of whole bacteria and with previously documented *in vitro* protective capacity with human cells. The immmunoproteomic component of our study is therefore based on common physicochemical features shared by all protein surfaces including those engaged in antigen-antibody interactions. Furthermore, the advantage of studying *Moraxella* cross-reactivity using mouse sera is connected with the fact that all antibodies specific against *M. catarrhalis* antigens in mouse are produced *de novo* without any interference from pre-existing antibodies. Nevertheless, since *M. catarrhalis* is a human specific pathogen, limitations of the mouse models have to be taken into account. In this context the relevance of our study to natural infections of human host should be confirmed in order to verify that our observed results mimic the human host response.

## Material and Methods

### Reagents

Acetonitrile (Sigma), Antibodies: peroxidase (HRP)-conjugated affinity pure goat anti-mouse IgG Fcγ-specific, (Jackson ImmunoResearch); bovine serum albumin BSA (SERVA); Immobilon Western Chemiluminescent HRP Substrate (EMD Millipore Inc.), sodium thiocyanate NaSCN (Sigma); sequencing grade modified porcine trypsin (Promega), TMB substrate reagent (R&D).

### Bacterial strains and culture

Two *M. catarrhalis* strains were used in the experimental studies: Mc6 and Mc8. The bacterial strains were routinely cultivated in BHI (brain heart infusion) broth at 37 °C with orbital shaking (150 rpm) and their characteristics were described previously^26^.

### Outer membrane vesicles isolation

Outer membrane vesicles (OMVs) were isolated according to Schaar *et al*.^28^ with some modifications. Briefly, 18 h cultures of *M. catarrhalis* strains were diluted 50-fold in 500 ml Brain Heart Infusion (BHI) media and incubated at 37 °C for 16–18 h, shaking (150 rpm). The cultures were harvested by centrifugation (8000 rpm for 15 min at 4 °C). The supernatants were collected and passed through 0.22 µm pore size filter vacuum pump (Merck, Millipore). The filtrates were concentrated using 50 kDa Vivaspin centrifugal concentrators (Amicon ultra, Millipore) at 5000 × g for 30 min at 4 °C. The concentrated supernatants were subsequently pelleted overnight (100 000 × g, at 4 °C) in an ultracentrifuge (Beckman Coulter Optima). The pellets containing OMVs were re-suspended in 500 µl of sterile PBS buffer (pH 7.4), aliquoted and stored in −20 °C. The sterility of the OMV preparations was confirmed on BHI agar. The protein concentrations in OMVs preparations were measured using Qubit fluorimeter or Bradford assay (Sigma-Aldrich Inc.) and the quality of OMVs preparations was confirmed in 12% SDS-PAGE.

### 2-D gel electrophoresis of OMV proteins

Two-dimensional electrophoresis (2-DE) procedure was carried out as previously described^40^. Samples were solubilized in rehydration solution (8 M urea, 2 M thiourea, 4% (*w/v*) CHAPS, 1% (*v/v*) Triton X-100, 10 mM Tris, 65 mM DTT, 0.8% (*v/v*) IPG buffer (pH 3-11, GE Healthcare Inc.), 0.01% (*w/v*) bromophenol blue) to the final protein concentration of 1 mg/ml. IPG strips (7 cm, pH 3-11NL, GE Healthcare Inc.) were saturated overnight with 120 µl of this solution and subjected to isoelectric focusing in a GE Healthcare Ettan IPGphor 3 Isoelectric Focusing Unit (3 hours, total voltage of 7000 V). Subsequently, IPG strips were equilibrated for 20 minutes in reducing buffer (6 M urea, 50 mM Tris, 30% (*v/v*) glycerol, 2% (*w/v*) SDS, 2% (*w/v)* DTT) followed by 20 minutes in alkylating buffer (6 M urea, 50 mM Tris, 30% (*v/v*) glycerol, 2% (*w/v*) SDS, 2,5% (*w/v)* iodoacetamide). The second dimension was resolved on 12% 1 mm-thick SDS-PAGE mini gels, with the separation voltage of 130 V. For each experiment, a set of three or four identical gels was prepared. One gel was stained with Page Blue Coomassie staining solution (Fermentas Inc.) according to the manufacturer’s instructions. The other gels were prepared for the further immunoblotting.

### Immunoblotting

Gels were saturated in transfer buffer (25 mM Tris, 192 mM glycine, 20% (*v/v*) methanol) for 15 minutes. Proteins were transferred to PVDF membranes with a semi-dry Transphor unit (BioRad Inc.) at 330 mA for 50 minutes. PVDF membranes were subsequently blocked with 5% BSA solution in the TBST buffer (20 mM Tris-HCl, 150 mM NaCl, 0,05% (*v/v*) Tween 20, pH 7,5) for 1 hour at room temperature. The blots were probed overnight at 4 °C with post-immunized pooled murine antisera: one with homologous antiserum and two others with heterologous antisera, diluted 1:60,000 at TBST buffer containing 0.1% BSA. Membranes were then washed three times for 5 minutes with TBST buffer, incubated with HRP-conjugated goat anti-mouse Fcγ diluted 1: 100,000 in TBST buffer with 0.1% BSA for 1 hour at room temperature and washed again three times for 5 minutes with TBST buffer. Chemiluminescent detection was carried out with Immobilon Western Chemiluminescent HRP Substrate and exposed to Kodak photographic film. Films were digitized with G:BOX imaging system (Syngene Inc.) The signal intensities of individual antigen reactions were compared and scored semi-quantitatively as described previously^55^ with quartile modification. Briefly, the intensity of several spots could not be analyzed directly due to their overlapping and notable differences in size. Therefore, we firstly collected the equal sections of the primary images, containing whole spots or the middle parts of the spots (depending on whether the given spot was smaller or larger than the selected area). The procedure was repeated for any sections containing image background, in order to compare and eliminate differences in the overall saturation of the photographic films. Subsequently, the intensity of the spot samples was evaluated against the respective samples of the image background, using Image Studio Lite software (LiCor Inc.). Results were presented as percent values relative to the most intense spot.

### In gel trypsin digestion for LC MS/MS analysis

Gels were rinsed with HPLC-grade water. Excised spots were destained in 100 µl of 100 mM ammonium bicarbonate/acetonitrile solution for 30 minutes at room temperature and then washed with 500 µl of neat acetonitrile. Gel pieces were then covered with 10 ng/µl porcine trypsin solution in 10 mM ammonium bicarbonate/10% (v/v) acetonitrile and incubated for 2 h on ice followed by overnight incubation at 37 °C. After digestion, samples were centrifuged and supernatant aliquots were withdrawn, and stored at −20 °C until LC MS/MS analysis.

### Identification of immunodominant membrane proteins by cross-reactive antibodies

The gels were manually aligned on the top of blots by using selected OMP CD, OMP E and Cop B proteins as landmark proteins as they were clearly identified in Coomassie blue stained gels from both two strains. High sequence coverage in mass spectrometry for both the proteins was observed.

### Peptide identification by LC MS/MS analysis

The analyses were performed on an Ion Trap LC/MS/MS spectrometer (Agilent Technologies, Santa Clara, CA USA). The resulting peptide mass fingerprints and LC MS/MS fragmentation spectra were identified using the MASCOT (http://www.matrixscience.com) and BLAST engines^56^ searching *M. catarrhalis* protein databases.

### SDS-PAGE and Western blots

For resolution of high molecular weight proteins present in OMVs, the 6% SDS-PAGE stained with GelCode Blue Stain Reagent (Thermo Scientific, USA). Proteins were transferred at 20 V overnight at 4 °C to an PVDF membrane (Millipore, USA) using Towbin buffer. The membranes were then blocked for 2 h with PBS supplemented with Tween 20 (T-PBS) and 3% BSA (SERVA). After 3 washes with T-PBS, the membrane strips were incubated with homologous or heterologous murine antisera in T-PBS-1% BSA for 2 h at 37 °C. After subsequent 3 washes, membrane strips were incubated with HRP-conjugated goat anti-mouse IgG Fcγ diluted 1, 000 in T-PBS-1% BSA for 2 h at 37 °C. Detection of proteins on blot transfer was carried out using substrates: 4-chloro-1-naphthol and perhydrol.

### Immunization and preparation of mouse antisera

The antisera were obtained as previously described^26^. Briefly, groups of 5–7 BALB/c mice of approximately equal weight, at 8–10 weeks old were immunized intraperitoneally with heat inactivated *M. catarrhalis* in saline (5 × 10^7^ cfu dose^−1^) in 0.1 ml doses, three times, at 2-week intervals (on days 0, 14 and 28). Ten days after the last immunization, mice were terminally bled through the retro-orbital sinus. Sera from each group immunized with particular immunogen (Mc1, Mc6 or Mc8) were pooled, divided and stored in aliquots at −70 °C until use. All experimental protocols were approved by the Ethical Review Committee of University of Environmental and Life Sciences of Wroclaw, Poland. This study complied with the animal experimentation guidelines of the National Institutes of Health guide for the care and use of Laboratory animals (NIH Publications No. 8023, revised 1978).

### Quantitative and Avidity ELISA

The relative avidities (AI) of antibodies to the OMV antigens, were determined by ELISA as described previously^26^.

### Genome sequencing

Four *Moraxella* genomes were sequenced using the Illumina MiSeq platform (University of Northumbria, UK). A 2*250 bp paired-end library (Nextera XT sample prep) were utilized for each sample. The raw data was processed by trimming the library adapter sequences and quality filtering was applied using CLC genomics workbench v.7.5 (CLC Bio, Aarhus, Denmark). Quality reads below 0.05, along with reads shorter than 100 bp and with 2 or more ambiguities were removed (total values removed for strain: MC1 – 0.06%; MC5 – 16.91%, MC6 – 7.55%, MC8 – 0.05%). After trimming and filtering, the data were processed and the reads were mapped to the reference genomes. The processed reads were used for *de novo* assembly. A high-quality draft genome was assembled by closing the gaps between the scaffolded contigs of the reference assembly by contig stitching with *de novo* contigs. The four final draft genomes comprised from 3 to 5 contigs were grouped in one scaffold each. The final genome assemblies were deposited in GenBank under accession numbers CP010573, CP010900, CP010901 and CP010902. Their detailed characteristics is provided in Supplementary Table 1.

The sequence of a pseudogene SQ02_07885 in the Mc8 genome was verified by PCR analysis using the primers, F: gataagccgtataccgccattcatg, R: ctgtctgagtgttctcttgcgcccag. The obtained 1.2 kb product was resequenced (Genomed, Poland).

### Preparing sequence data for bioinformatics analysis

Four newly sequenced and all publicly available 23 full genomes from *M. catarrhalis* strains as well as four other *Moraxella* species were included in the bioinformatics analysis (Supplementary Table 2). Genomes that were not fully sequenced, assembled or annotated were excluded because they would bias the presence and distribution of genes. As an outgroup three representatives of *Acinetobacter* were selected because they are close relatives to *Moraxella*, grouped together to family Moraxellaceae. The data for these genomes were downloaded from the GenBank database (ftp.ncbi.nlm.nih.gov/genomes). Their annotated protein sequences were clustered into orthologous groups using a genome-scale algorithm OrthoMCL^57^ assuming BLAST cut-off for sequence similarity 25% and E-value threshold 10^−3^.

Phylogenetic relationships between the genomes studied were inferred using concatenated alignments of sequences from 739 orthologous groups that were represented by one sequence in each of all 34 genomes. The sequences were aligned with a slow and accurate algorithm PSI-Coffee, which combines homology extension and consistency based progressive alignment^58^. Sites appropriate for phylogenetic analyses were selected in Gblocks using less stringent criteria^59^. In the final analyses, the alignment with 235,616 sites was used.

### Searching for domains in protein sequences and their classification to functional categories

The presence of protein domains in OMV proteins were analyzed by local searches of Conserved Domain Database, CDD^60^ using rpsBLAST assuming E-value < 0.01. Protein sequences from the studied genomes were classified to more general functional categories according to Clusters of Orthologous Groups (COGs) being a part of the CDD database.

### Inferring phylogenetic relationships based on orthologous sequences

In the construction of phylogenetic trees, a Bayesian approach was applied with MrBayes^61^ and maximum likelihood method in morePhyML^62^ using PhyML^63^. In the MrBayes analysis, a mixed + Γ + I model of amino acid substitutions was used to specify appropriate models across the larger space in the Bayesian MCMC analysis^64^, avoiding the need for *a priori* model testing. In this approach, two independent runs starting from random trees, each using eight Markov chains were applied. Trees were sampled every 100 generations for 1,000,000 generations. In the final analysis, trees from the last 703,000 generations that reached the stationary phase and convergence (i.e. the standard deviation of split frequencies stabilized and was lower than the proposed threshold of 0.01) were selected. The best-fit substitution model LG + F + Γ + I found among all 120 candidates in ProtTest^65^ and the best search algorithm NNI + SPR were applied in morePhyML. To assess the significance of the branches, non-parametric bootstrap analysis was performed on 1000 replicates in PhyML.

The percentage difference between sequences of OMV proteins and the rest of orthologous groups, as well as homologous sequences of proteins and their epitope sites, analyed in cross-reaction studies was calculated using ClustalW (Larkin *et al*. 2007) for alignments obtained in PSI-Coffee. To avoid a bias resulting from the presence and absence of proteins in different phylogenetic lineages, only sequences represented in all 34 genomes in the comparison of OMV and other orthologous protein groups were analysed.

### Inferring phylogenetic relationships based on presence/absence of genes

Phylogenetic relationships were calculated based on the presence and absence of 4780 orthologous groups found by OrthoMCL in least two genomes. The presence of some genes only in 28 genomes were also included in the dataset. Two methods were applied, Bayesian in MrBayes^61^ and maximum parsimony in PAUP*^66^. The characters were considered as unordered. In MrBayes approach, the gamma-distributed rate model and two independent runs starting from random trees using 4 Markov chains were applied. Trees were sampled every 100 generations for 10,000,000 generations. In the final analysis, trees from the last 2,716,000 generations that reached the stationary phase and convergence were selected. In PAUP*, the final tree was searched from 10 starting trees obtained by stepwise addition of characters’ sequences followed by the tree-bisection-reconnection (TBR) branch-swapping algorithm. In the non-parametric bootstrap analyses, we assumed 1000 replicates.

### Inferring ancestral sizes of gene content

Ancestral sizes of gene content in the genomes as well as gene gains and losses per phylogenetic lineage were determined in BadiRate^67^ under the free rates birth-death-and-innovation model and maximum likelihood estimation procedure. Phylogenetic relationships were assumed as deduced in the phylogenetic analysis in MrBayes, whereas data about the size of homologous protein families in the studied genomes were taken from OrthoMCL results.

### Computational prediction of potential epitope sites

Sequences of immunoreactive proteins were searched for potential epitopes using 15 predictive approaches: AAP^68^, BCPred^69^, BepiPred^70^, CBTOPE^71^, COBEpro^72^, FBCPred^73^, IgPred^74^, SVMTrip^75^, as well as BcePred^76^ based on seven amino acid physico-chemical scales, hydrophilicity, flexibility, accessibility, turns, antigenic propensity, exposed residues and polarity. These results were summarized by counting the number of times individual sites in the sequences were predicted as an epitope^77^.

### Statistical analyses

Statistical analyses were carried out in R package Statistical significance between the numbers of protein sequences in functional categories was estimated using likelihood ratio (G-test) test of independence and test of proportions. P-values were corrected with Benjamini–Hochberg procedure for multiple testing. Differences in contribution of predicted epitope sites in sequences of proteins showing various cross-reactivity were analysed in t-test.

### Data Availability

The genome sequences of Mc1, Mc5, Mc6 and Mc8 can be found in GenBank under accession numbers: CP010573, CP010900, CP010901, and CP010902 respectively. Most data generated or analyzed during this study are included in this published article (and its Supplementary Information files). Other data supporting this study are available from the corresponding author upon reasonable request.

## Electronic supplementary material

Supplementary Information

Data S1

Data S2

Data S3

Data S4
