# Supplementary material for: Virulence factors of Moraxella catarrhalis outer membrane vesicles are major targets for cross-reactive antibodies and have adapted during evolution
Source: Sci Rep. 2018 Mar 21;8:4955. doi: 10.1038/s41598-018-23029-7 (PMC5862889; doi:10.1038/s41598-018-23029-7)
Supplement: Supplementary file 1 — Supplementary Information [file 41598_2018_23029_MOESM1_ESM.pdf]

Supplementary File: Tables and Figures

Virulence factors of *Moraxella catarrhalis* outer membrane vesicles are major targets for cross-reactive antibodies and have adapted during evolution

Daria Augustyniak[a]*, Rafał Seredyński[b,c], Siobhán McClean[d], Justyna Roszkowiak[a], Bartosz Roszniowski[a], Darren L. Smith[e], Zuzanna Drulis-Kawa[a], Paweł Mackiewicz[f]*

(a) Department of Pathogen Biology and Immunology, Institute of Genetics and Microbiology, University of Wroclaw, Przybyszewskiego 63/77, 51-148 Wroclaw, Poland

E mail: daria.augustyniak@uwr.edu.pl

Email: justyna.roszkowiak@uwr.edu.pl

Email: bartosz.roszniowski@uwr.edu.pl

Email: zuzanna.drulis-kawa@uwr.edu.pl

(b) Department of Physiology, Wroclaw Medical University, T. Chalubinskiego 10, 50-368 Wroclaw, Poland

(c) Department of Physical Chemistry of Microorganisms, Institute of Genetics and Microbiology, University of Wroclaw, Przybyszewskiego 63/77, 51-148 Wroclaw, Poland

E-mail: rafal.seredynski@umed.wroc.pl

(d) School of Biomolecular and Biomedical Sciences, UCD O'Brien Centre for Science West B304, Dublin, Ireland

E-mail: siobhan.mcclean@ucd.ie

(e) Applied Sciences, University of Northumbria, Ellison Building EBD222, Newcastle upon Tyne NE1 8ST, UK

E-mail: darren.smith@northumbria.ac.uk

(f) Department of Genomics, Faculty of Biotechnology, University of Wrocław, Joliot-Curie 14a, 50-383 Wrocław, Poland

E-mail: pamac@smorfland.uni.wroc.pl

∗ Corresponding authors:

daria.augustyniak@uwr.edu.pl, Tel: +48 71 37 56 296 (D. Augustyniak)

pamac@smorfland.uni.wroc.pl, Tel: +48 71 37 56 331 (P. Mackiewicz)

Supplementary Table S1. Characteristics of newly sequenced genomes from four *M. catarrhalis* strains.

Characteristics	*Moraxella catarrhalis* genome			
	**Mc1**	**Mc5**	**Mc6**	**Mc8**
Accession number	CP010573	CP010900	CP010901	CP010902
Total % of trimmed contigs	0.06	16.91	7.55	0.05
Total bp after trimming	952,873,918	1,060,357,667	958,836,852	954,456,191
Total reads after trimming	4,777,572	5,014,740	4,393,940	5,698,340
Average read length	199.4	211.4	219.9	289.5
Reference genome accession number	CP002005.1	NC_014147.1	CP007669.1	MC1
De novo assembly contigs	256	113	395	251
Average contig size	7,983	5,621	5,309	7,946
N50 contig	90,957	98,344	78,381	93,356
Largest contig	215,285	174,662	279,058	323,133
Number of contigs after stitching	5	4	3	4
Total length (bp) of genome	1,843,517	1,852,323	1,839,052	1,849,375
Number of genes	1699	1710	1683	1696
GC% content	41.7	41.7	41.8	41.8

Supplementary Table S2. *Moraxella* and *Acinetobacter* genomes used in the study. The number of all annotated protein-coding genes as well as genes classified into orthologous groups by OrthoMCL and ungrouped genes are shown.

Organism	Accession number	Total protein-coding genes	Genes in orthologous groups	Ungrouped genes
Acinetobacter calcoacaticus PHEA-2	GCF000191145.1	3599	3302	297
Acinetobacter oleivorans DR1	GCF000196795.1	3786	3415	371
Acinetobacter sp. 114	GCF001307195.1	2679	2245	434
Moraxella boevrei DSM 14165	GCF000379845.1	2220	1934	286
Moraxella bovoculi 237	GCF000696305.1	1820	1640	180
Moraxella caprae DSM 19149	GCF000426885.1	2320	1897	423
Moraxella catarrhalis 101P30B1	GCF000193065.1	1634	1633	1
Moraxella catarrhalis 103P14B1	GCF000192925.1	1709	1696	13
Moraxella catarrhalis 12P80B1	GCF000192965.1	1727	1562	165
Moraxella catarrhalis 157.rep1_MCAT	GCF001062845.1	1707	1707	0
Moraxella catarrhalis 157.rep2_MCAT	GCF001062815.1	1709	1709	0
Moraxella catarrhalis 25240	GCF000740455.1	1678	1675	3
Moraxella catarrhalis 304_MCAT	GCF001064405.1	1766	1764	2
Moraxella catarrhalis 324_MCAT	GCF001064535.1	1769	1765	4
Moraxella catarrhalis 339_MCAT	GCF001064655.1	1657	1654	3
Moraxella catarrhalis 340_MCAT	GCF001064665.1	1652	1652	0
Moraxella catarrhalis 46P47B1	GCF000192945.1	1629	1625	4
Moraxella catarrhalis 489_MCAT	GCF001063475.1	1583	1583	0
Moraxella catarrhalis 611_MCAT	GCF001065625.1	1598	1597	1
Moraxella catarrhalis 613_MCAT	GCF001065675.1	1591	1590	1
Moraxella catarrhalis 7169	GCF000192905.1	1688	1686	2
Moraxella catarrhalis ATCC 25239	GCF000766665.1	1639	1637	2
Moraxella catarrhalis BBH18	GCF000092265.1	1621	1620	1
Moraxella catarrhalis BC1	GCF000192985.1	1700	1697	3
Moraxella catarrhalis BC7	GCF000193005.1	1675	1649	26
Moraxella catarrhalis BC8	GCF000193025.1	1681	1676	5
Moraxella catarrhalis CO72	GCF000193045.1	1728	1722	6
Moraxella catarrhalis MC1	CP010573.1	1589	1588	1
Moraxella catarrhalis MC5	CP010900.1	1604	1604	0
Moraxella catarrhalis MC6	CP010901.1	1581	1579	2
Moraxella catarrhalis MC8	CP010902.1	1580	1578	2
Moraxella catarrhalis O35E	GCF000193085.1	1544	1544	0
Moraxella catarrhalis RH4	GCF000302495.1	1609	1605	4
Moraxella macacae 0408225	GCF000320365.1	1805	1691	114

Supplementary Table S3. Percentage of OMV proteins and other proteins from *Moraxella catarrhalis* in their given functional categories of Cluster of Orthologous Groups (COGs). P-value of proportion test comparing the number of proteins was corrected by Benjamini–Hochberg procedure. In total 1673 such OMV proteins were found with 48,621 other proteins.

Functional category	OMVs	non-OMV	P-value
Amino acid transport and metabolism	5.56	9.20	9.0887E-07
Carbohydrate transport and metabolism	4.06	2.24	2.86218E-06
Cell cycle control, cell division, chromosome partitioning / Cytoskeleton	3.53	1.54	1.02204E-09
Cell wall/membrane/envelope biogenesis	14.94	6.04	1.96064E-47
Cell motility and secretion	0.00	0.84	0.0004
Chromatin structure and dynamics	0.00	0.06	0.6121
Coenzyme transport and metabolism	0.00	7.14	1.27593E-28
Defence mechanisms	0.00	1.66	4.42432E-07
DNA replication, recombination and repair	0.12	6.51	3.75724E-25
Energy production and conversion	5.92	7.37	0.0361
Extracellular structures	1.49	0.00	4.9528E-146
Function unknown	7.47	8.32	0.2773
General function prediction only	11.06	10.15	0.2773
Inorganic ion transport and metabolism	10.46	5.40	5.11772E-18
Intracellular trafficking, secretion and vesicular transport	3.53	3.05	0.3271
Lipid transport and metabolism	2.03	3.46	0.0028
Nucleotide transport and metabolism	0.00	3.47	5.18827E-14
Posttranslational modification, protein turnover, chaperones	8.31	5.72	1.89006E-05
RNA processing and modification	0.00	0.07	0.5552
Secondary metabolites biosynthesis, transport and catabolism	3.77	1.27	3.05896E-17
Signal transduction mechanisms	0.00	2.35	1.02204E-09
Transcription	5.74	3.92	0.0004
Translation, ribosomal structure and biogenesis	12.01	10.22	0.0264

Supplementary Table S4. Percentage of annotated protein sequences unique for *Moraxella catarrhalis* (Mcat) and unique for other *Moraxella* species (Msp) in given functional categories of Cluster of Orthologous Groups (COGs). P-value of proportion test comparing the number of proteins was corrected by Benjamini–Hochberg procedure. In total 2611 such proteins were found in *Moraxella catarrhalis* and 2162 in other *Moraxella* species.

Functional category	Mcat	Msp	P-value
Amino acid transport and metabolism	14.82	5.41	1.13E-24
Carbohydrate transport and metabolism	1.00	4.35	1.31E-12
Cell cycle control, cell division, chromosome partitioning / Cytoskeleton	0.23	2.08	3.93E-09
Cell wall/membrane/envelope biogenesis	4.90	8.37	3.67E-06
Cell motility and secretion	0.61	6.52	2.52E-28
Chromatin structure and dynamics	0.00	0.05	0.9687
Coenzyme transport and metabolism	7.35	1.57	1.03E-19
Defence mechanisms	7.77	2.54	1.32E-14
DNA replication, recombination and repair	3.56	5.13	0.0146
Energy production and conversion	1.38	2.08	0.0962
Extracellular structures	0.04	0.00	1.0000
Function unknown	10.88	12.63	0.0872
General function prediction only	17.85	13.41	7.05E-05
Inorganic ion transport and metabolism	9.65	5.55	4.69E-07
Intracellular trafficking, secretion and vesicular transport	1.88	7.45	1.03E-19
Lipid transport and metabolism	4.14	2.73	0.0156
Nucleotide transport and metabolism	1.42	0.46	0.0025
Posttranslational modification, protein turnover, chaperones	1.42	2.17	0.0848
Secondary metabolites biosynthesis, transport and catabolism	1.26	2.64	0.0014
Signal transduction mechanisms	3.45	7.91	7.22E-11
Transcription	6.01	6.34	0.7560
Translation, ribosomal structure and biogenesis	0.38	0.60	0.4424

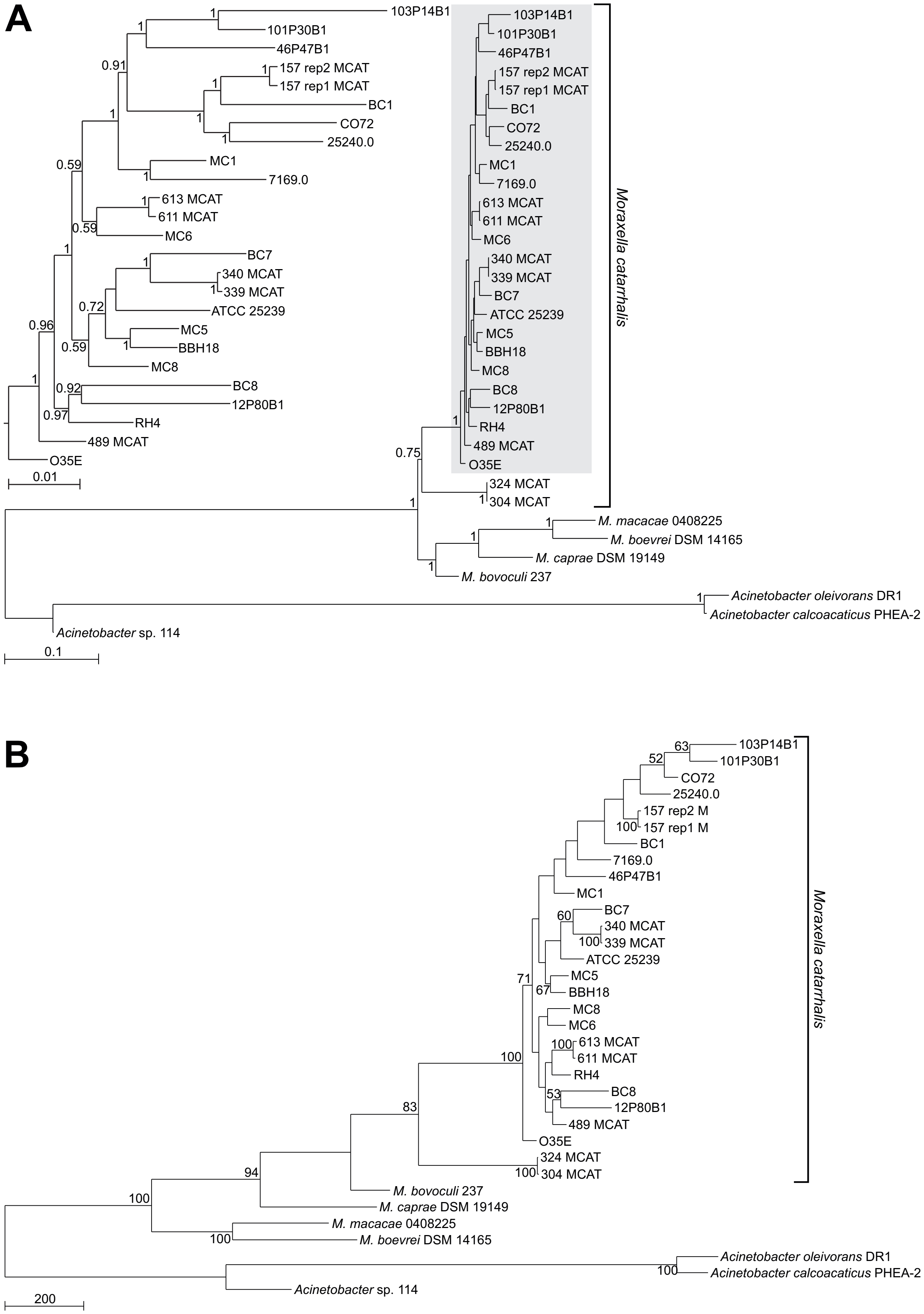

Supplementary Fig. S1. Trees based on the presence and absence of orthologous groups of proteins found by OrthoMCL in 31 *Moraxella* genomes and three members of *Acinetobacter* genus. A. The Bayesian tree constructed in MrBayes. The part of tree marked by the grey rectangle is shown on the left in the larger scale. Numbers at nodes correspond to posterior probabilities. Values larger than 0.5 are only shown. B. Maximum parsimony tree found in PAUP*. Numbers at nodes correspond to bootstrap values. Only values larger than 50% are shown.

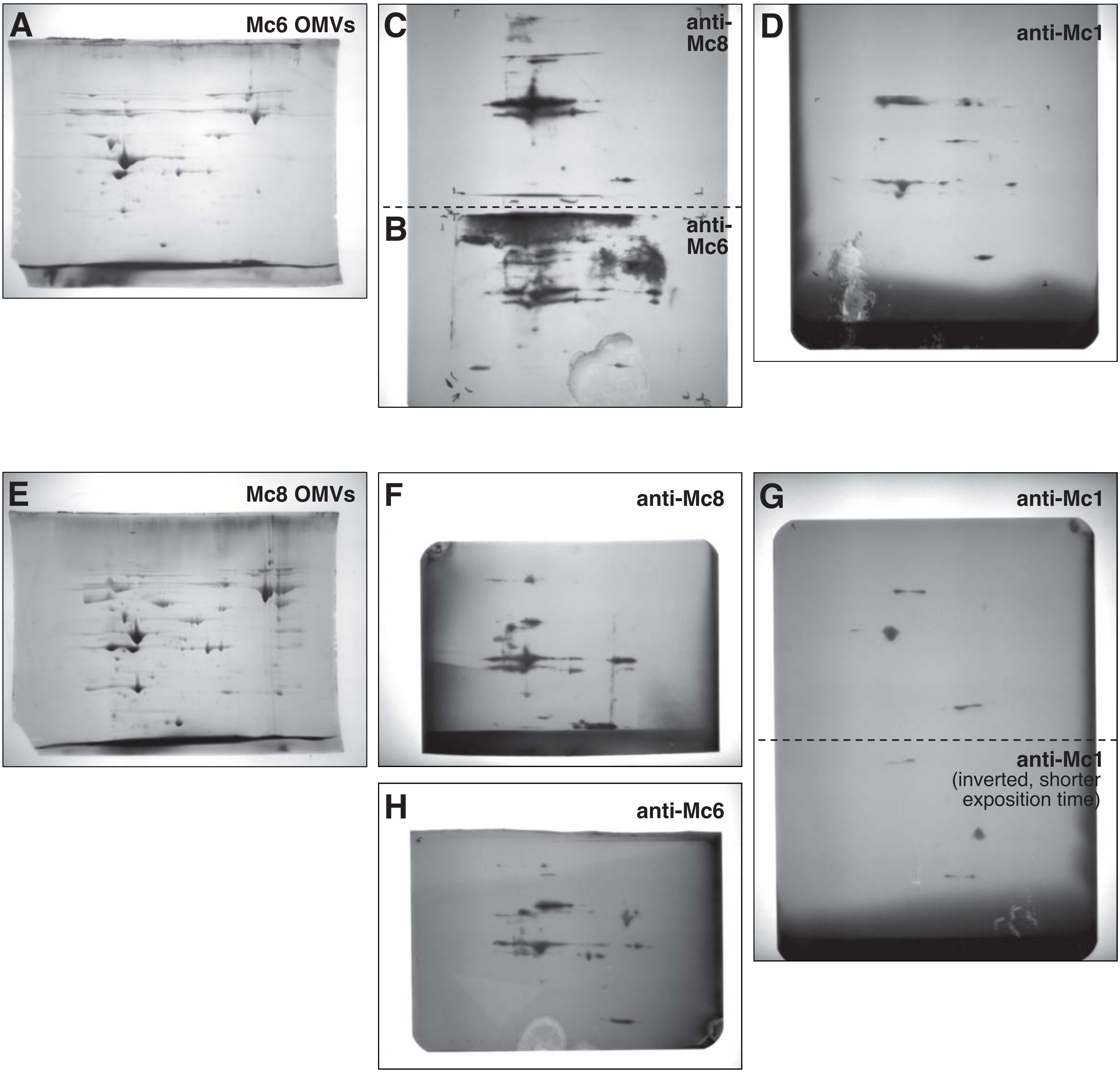

Supplementary Fig. S2. Original images of two-dimensional gels and blots, acquired with G:BOX system (Syngene Inc.); all the X-ray films (B-D, F, G and the upper part of H) were exposed to the chemiluminescent substrate for the exactly same time. Immunoblot images were used for semi-quantitative intensity analysis. Cropped versions with adjusted brightness and contrast are included in the Figure 2. (A, E) 2D-gel electrophoresis OMV proteins visualized by Coomassie staining; (B, F) 2D immunoblots probed with homologous antisera; (C, G) immunoblots probed with stronger cross-reactive antisera; (D, H) immunoblots probed with weaker cross-reactive antisera.

A

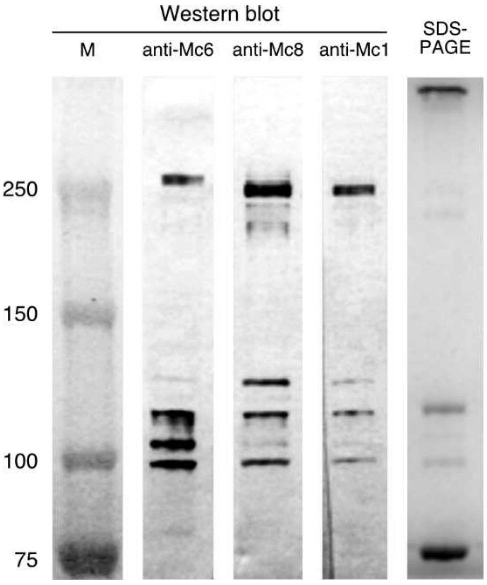

Original Western blot image

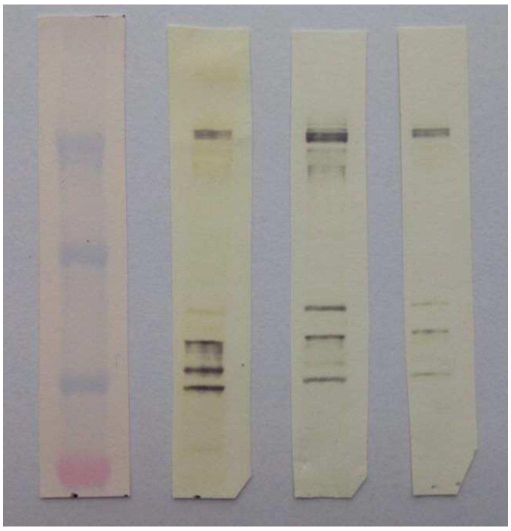

B

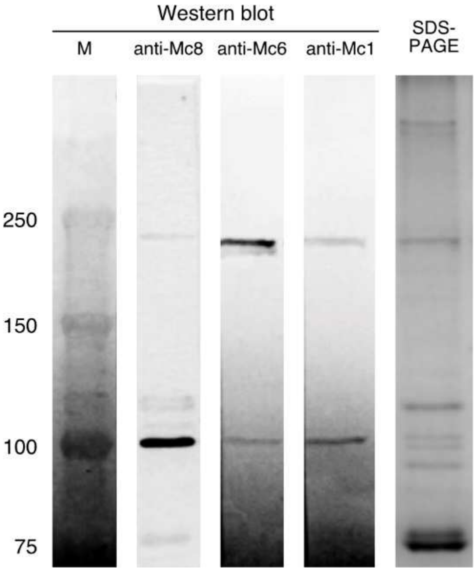

Original Western blot image

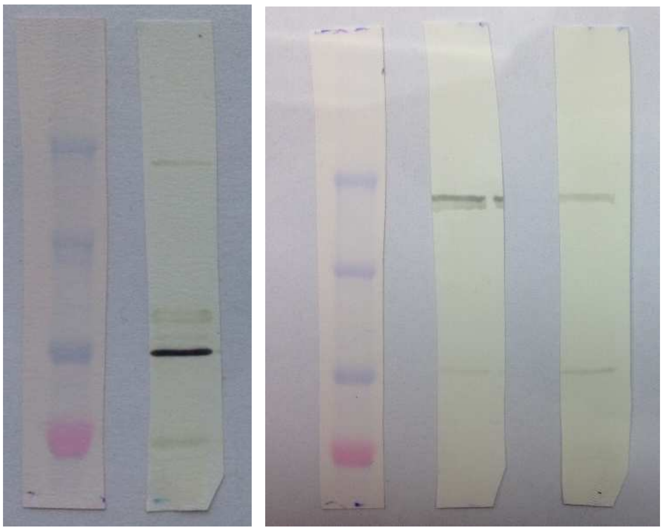

C

Mc6 OMVs SDS-PAGE after transfer

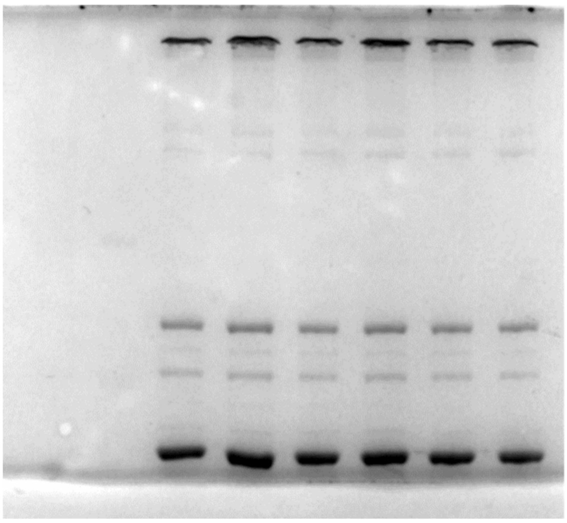

Mc8 OMVs SDS-PAGE after transfer

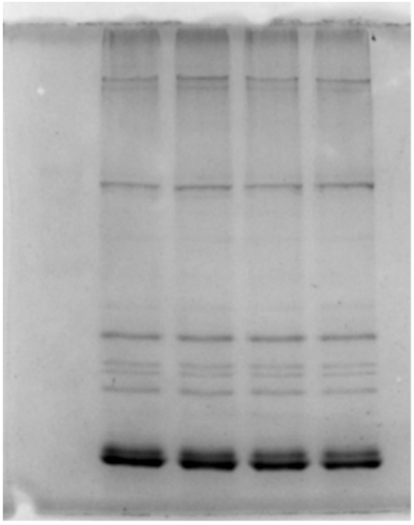

D

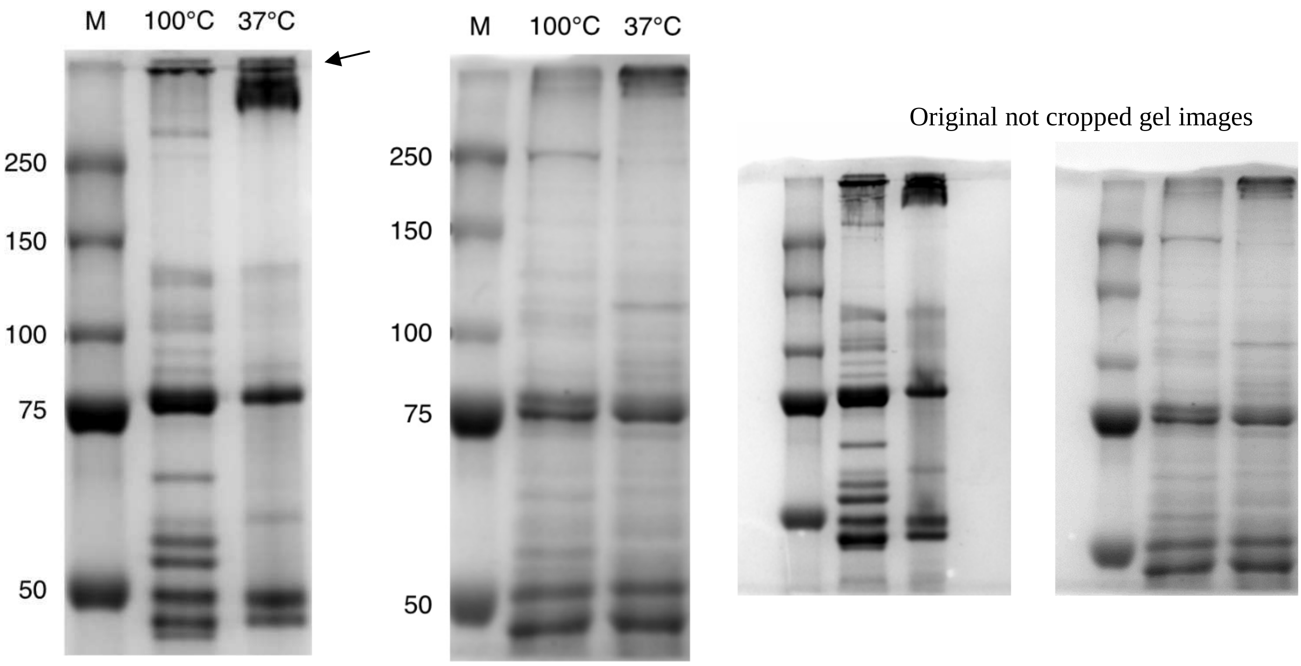

Supplementary Fig. S3. Analyses of high molecular weight antigens (HMW) carried by *Moraxella catarrhalis* OMVs. Western blot-based detection of HMW antigens of Mc6 OMVs (A) and Mc8 OMVs (B). Equal amount of proteins were resolved by 6% SDS-PAGE and analysed using indicated murine antisera and anti-Fcγ conjugate. The strong reactivity for ~ 250 kDa HMW antigen, presumably Hag/MID is shown. The color blots refer to original images before white and black adjustements. Molecular markers in kDa are shown on stained immobilon P on the left side of the blot set. (C) original not cropped 6% SDS-PAGE gels of Mc6 OMVs (left panel) and Mc8 OMVs (right panel), after transfer stained with Coomassie (D) 8% SDS-PAGE of OMVs Mc6 (left panel) and OMVs Mc8 (middle panel) heated for 10 min in indicated temperatures to show the presence of HMW antigen (indicated by arrow) and its resistance to heating that is typical for UspA2, only for Mc6. The original not cropped gel images are presented in the right panel.

CopB: MC1_SP69_02275

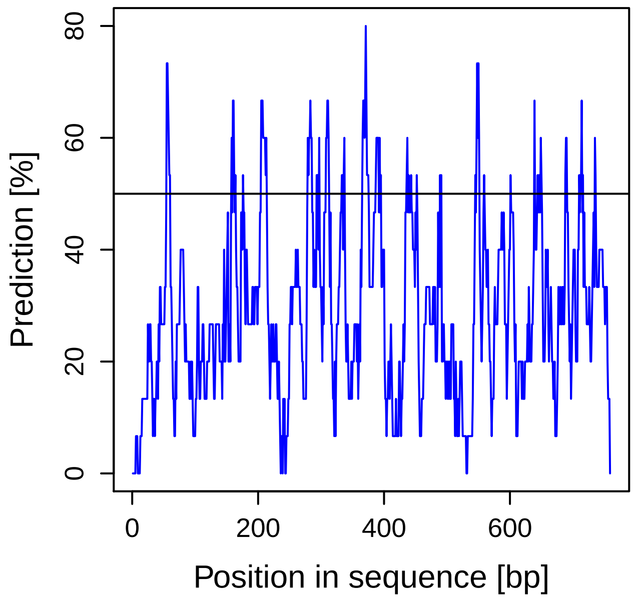

CopB: MC6_SQ01_06490

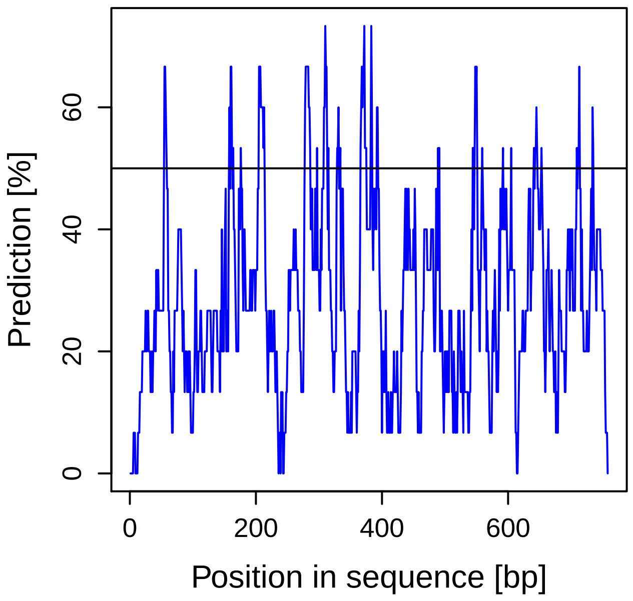

CopB: MC8_SQ02_06550

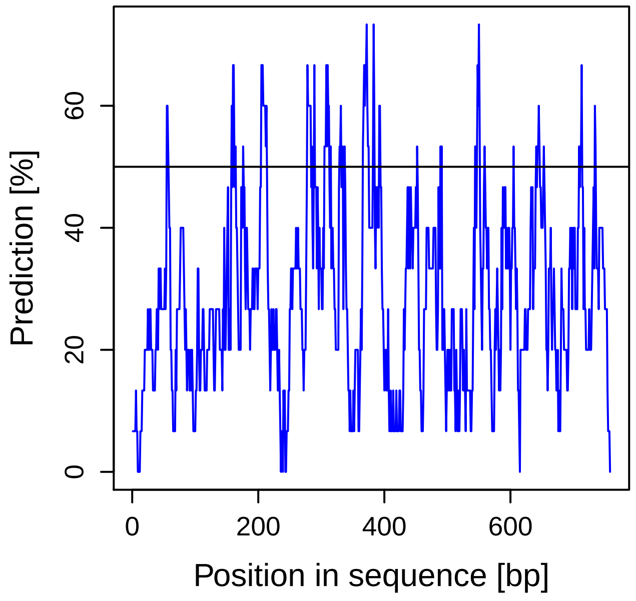

FbpA: MC1_SP69_01350, MC8_SQ02_07515

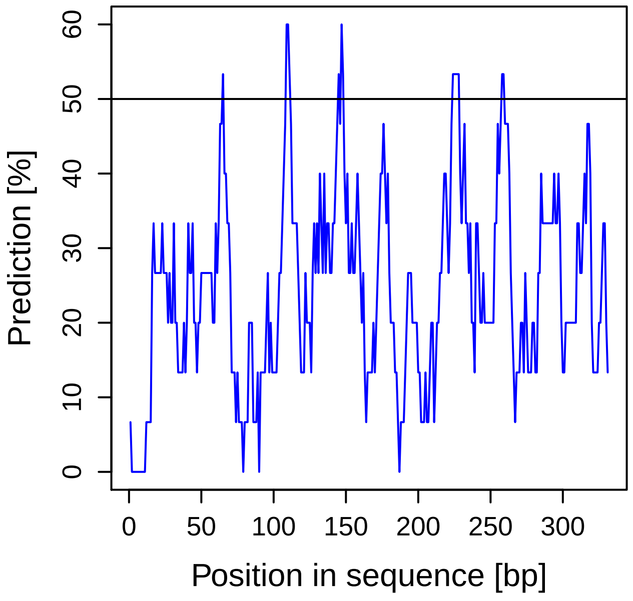

FbpA: MC6_SQ01_07425

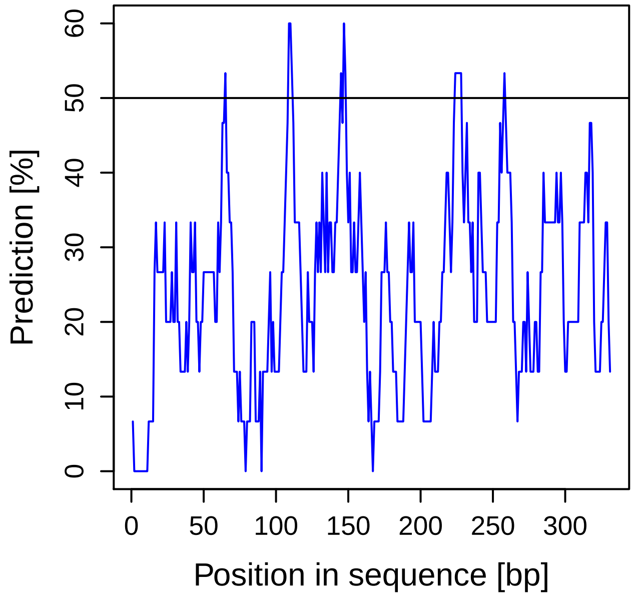

GroEL: MC1_SP69_06775, MC6_SQ01_02125

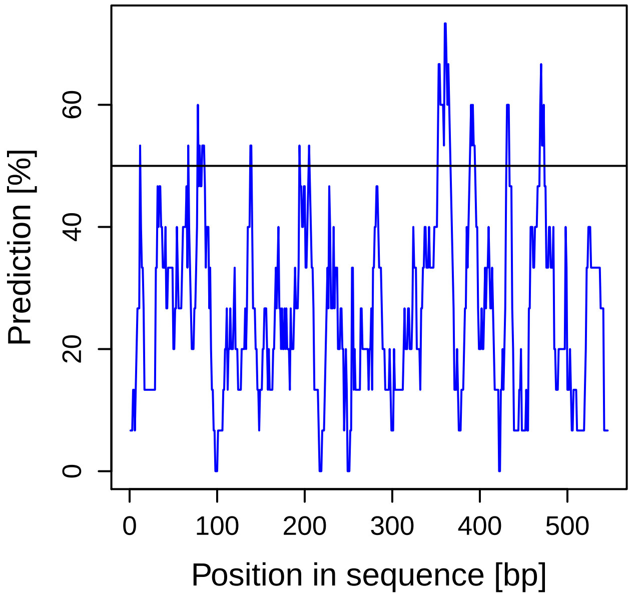

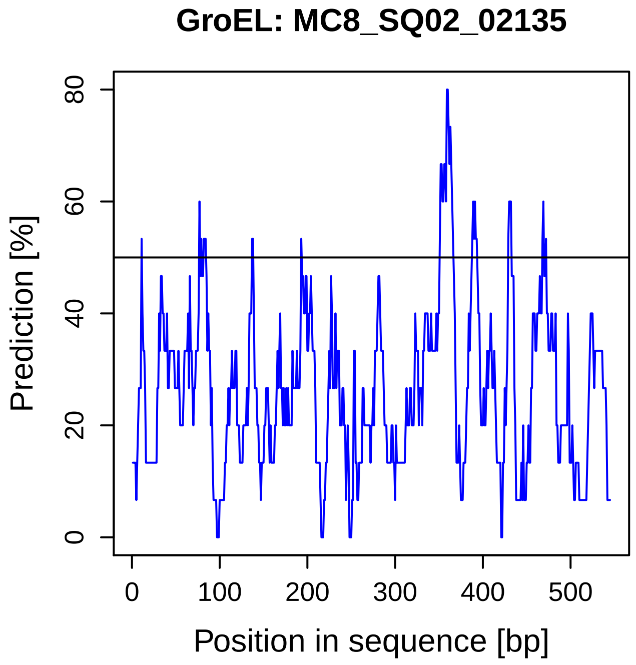
GroEL: MC8_SQ02_02135
Prediction [%]
0
20
40
60
80
0
100
200
300
400
500
Position in sequence [bp]

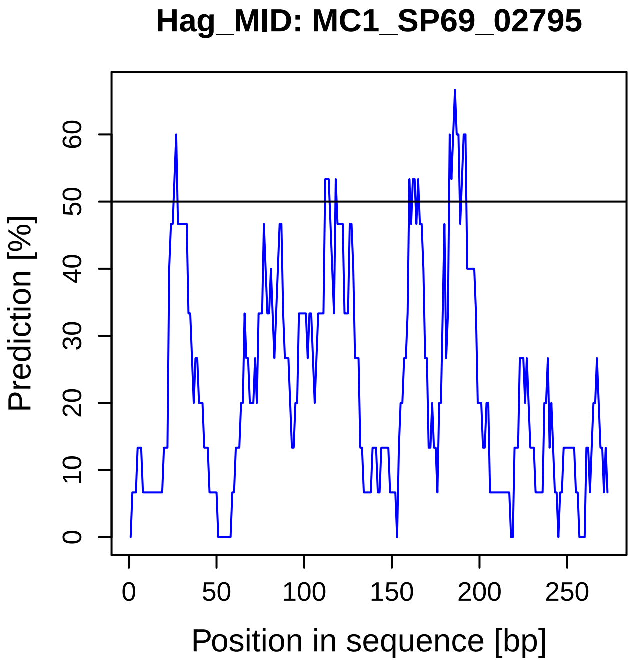
Hag_MID: MC1_SP69_02795
Prediction [%]
0
10
20
30
40
50
60
0
50
100
150
200
250
Position in sequence [bp]

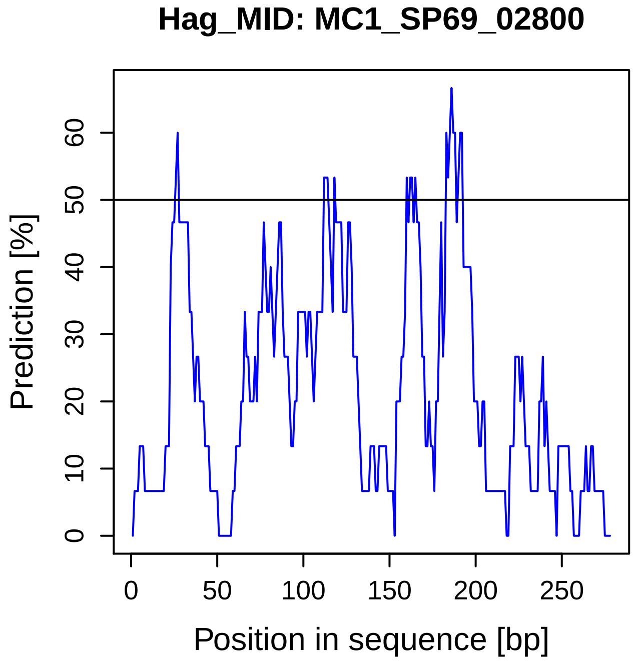
Hag_MID: MC1_SP69_02800
Prediction [%]
0
10
20
30
40
50
60
0
50
100
150
200
250
Position in sequence [bp]

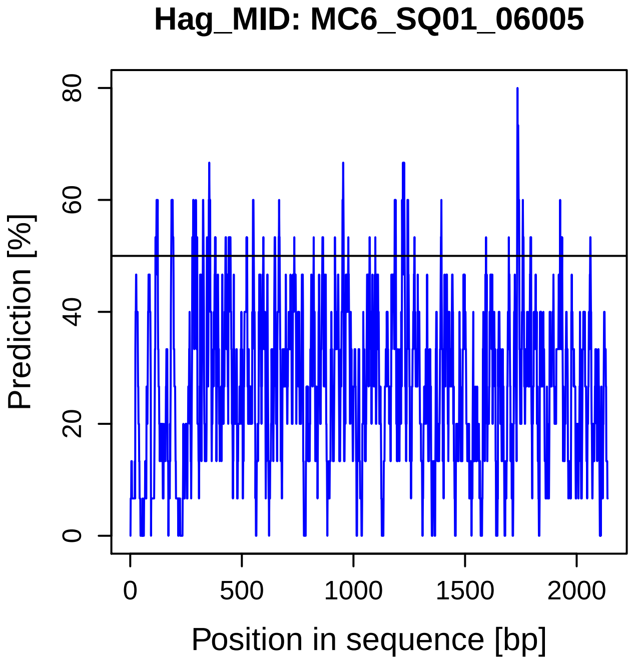
Hag_MID: MC6_SQ01_06005
Prediction [%]
0
20
40
60
80
0
500
1000
1500
2000
Position in sequence [bp]

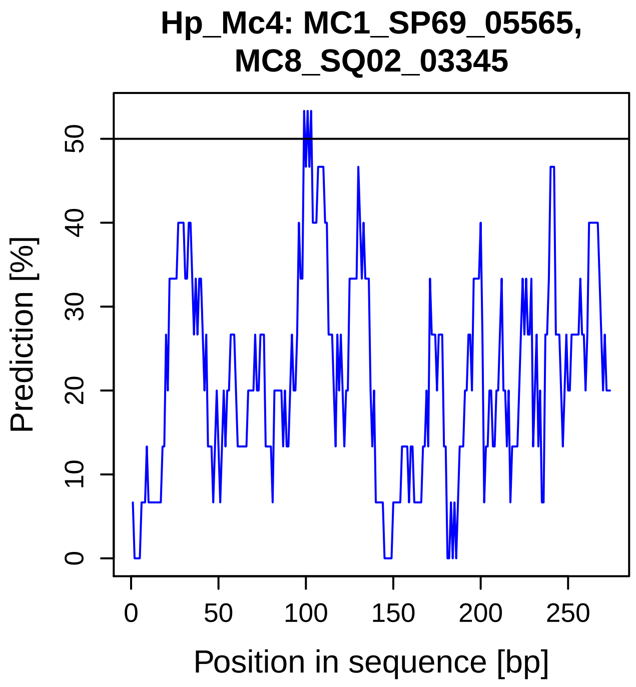
Hp_Mc4: MC1_SP69_05565,
MC8_SQ02_03345
Prediction [%]
0
10
20
30
40
50
0
50
100
150
200
250
Position in sequence [bp]

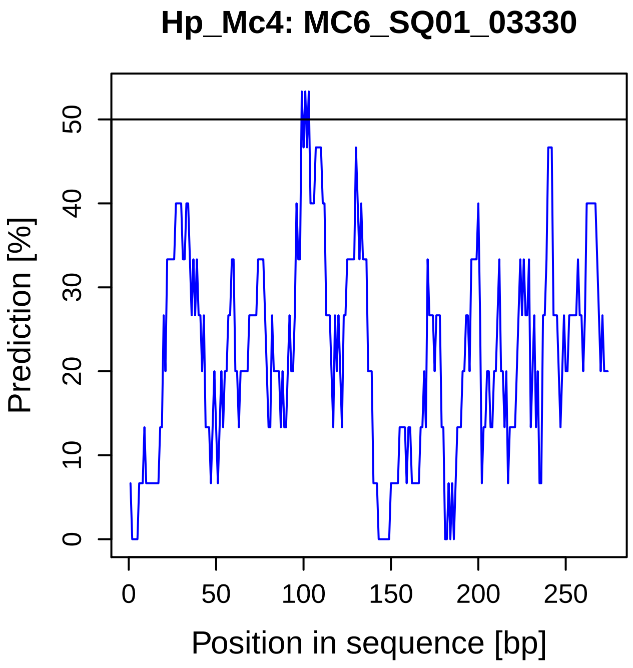
Hp_Mc4: MC6_SQ01_03330
Prediction [%]
0
10
20
30
40
50
0
50
100
150
200
250
Position in sequence [bp]

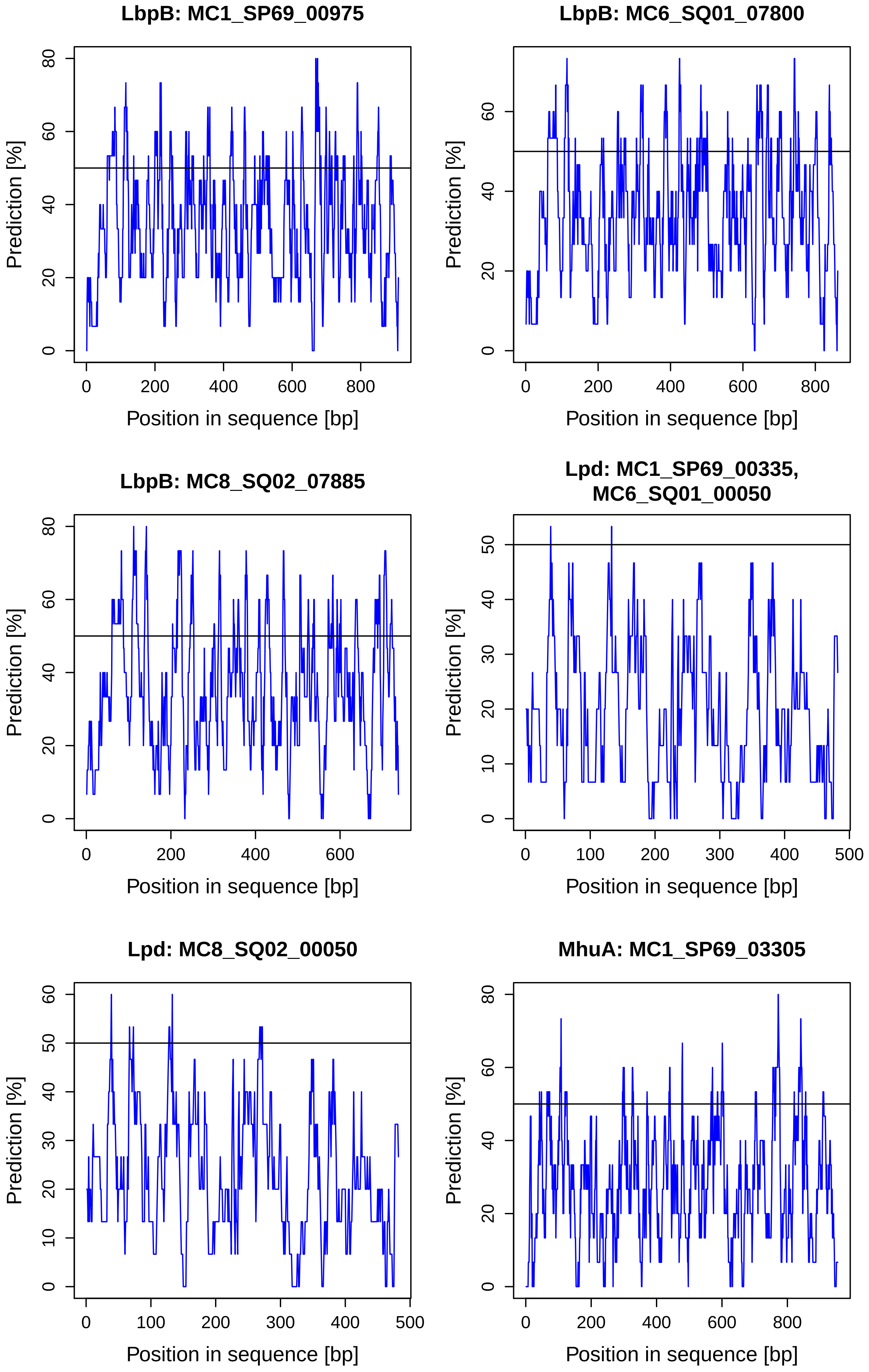
LbpB: MC1_SP69_00975
LbpB: MC6_SQ01_07800
LbpB: MC8_SQ02_07885
Lpd: MC1_SP69_00335, MC6_SQ01_00050
Lpd: MC8_SQ02_00050
MhuA: MC1_SP69_03305
Prediction [%]
Position in sequence [bp]

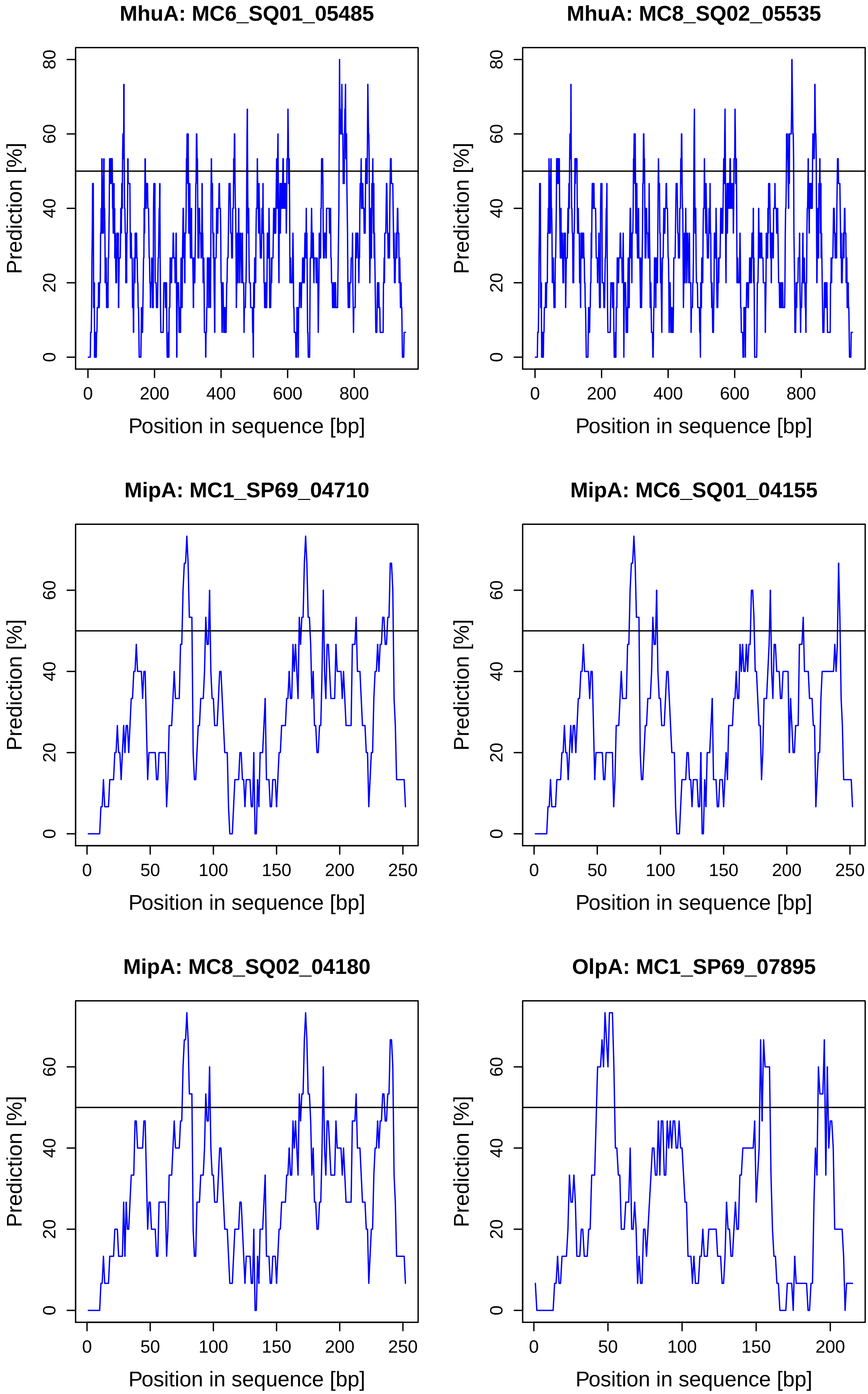
MhuA: MC6_SQ01_05485
MhuA: MC8_SQ02_05535
MipA: MC1_SP69_04710
MipA: MC6_SQ01_04155
MipA: MC8_SQ02_04180
OlpA: MC1_SP69_07895
Prediction [%]
Position in sequence [bp]

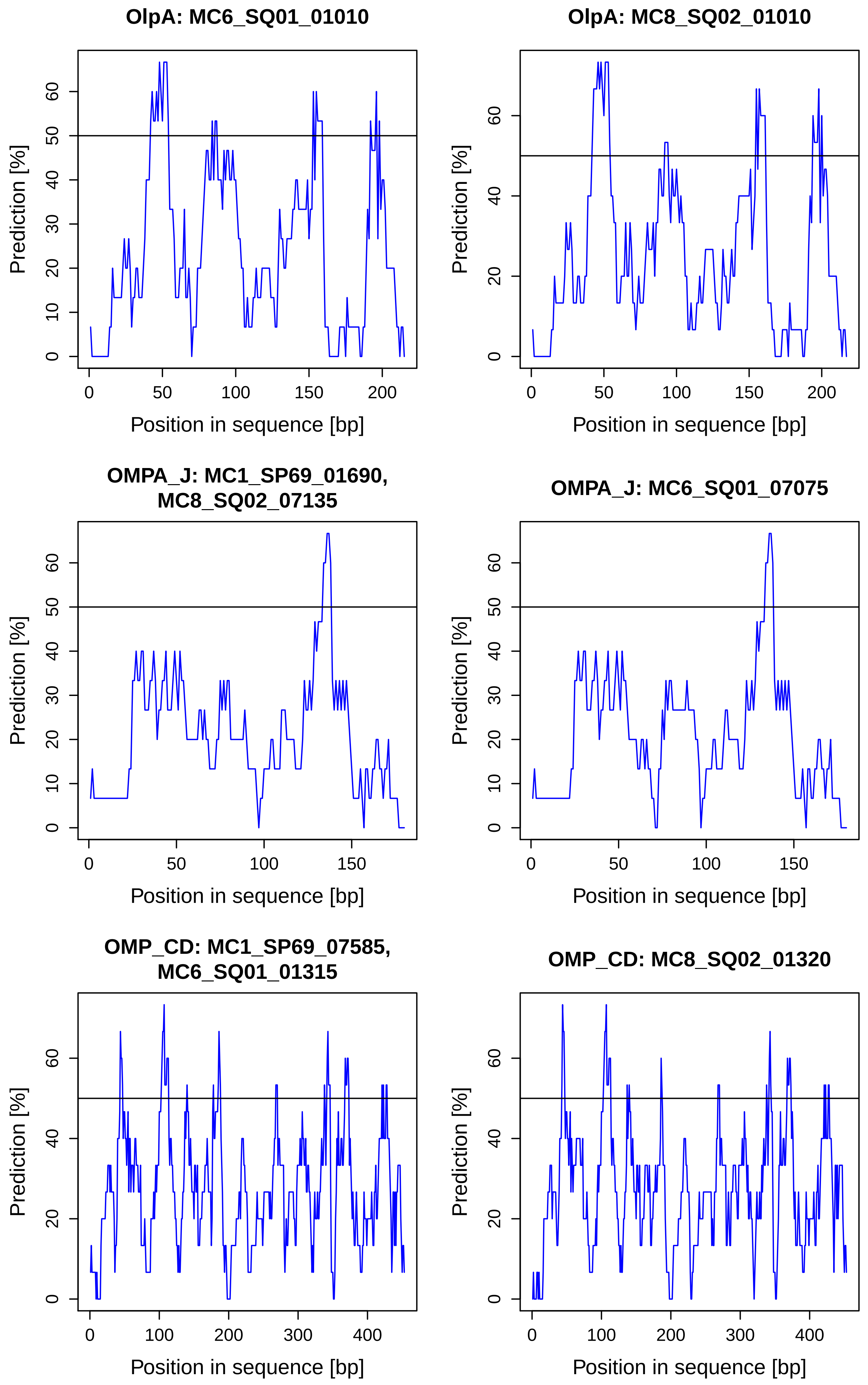
OlpA: MC6_SQ01_01010
OlpA: MC8_SQ02_01010
OMPA_J: MC1_SP69_01690, MC8_SQ02_07135
OMPA_J: MC6_SQ01_07075
OMP_CD: MC1_SP69_07585, MC6_SQ01_01315
OMP_CD: MC8_SQ02_01320
Prediction [%]
Position in sequence [bp]

OMP_E: MC1_SP69_03870, MC6_SQ01_04940

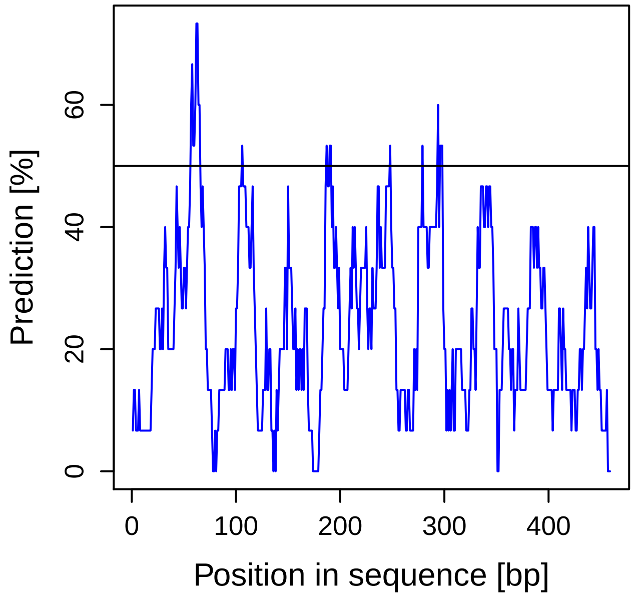

OMP_E: MC8_SQ02_04980

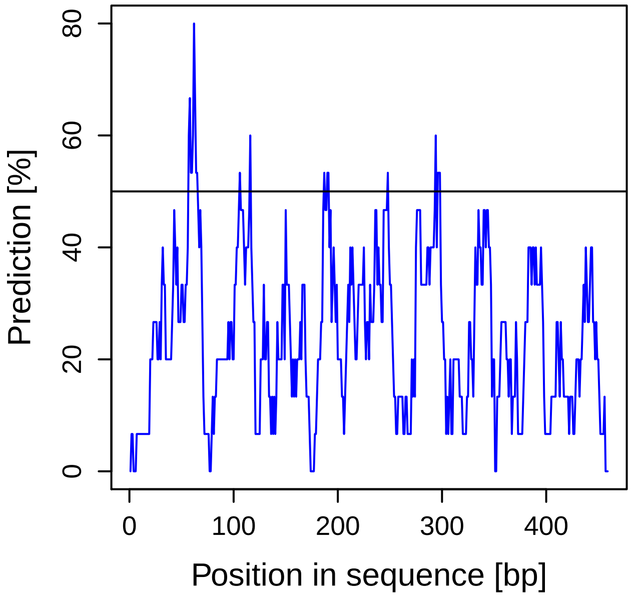

OMP_G1b: MC1_SP69_06250

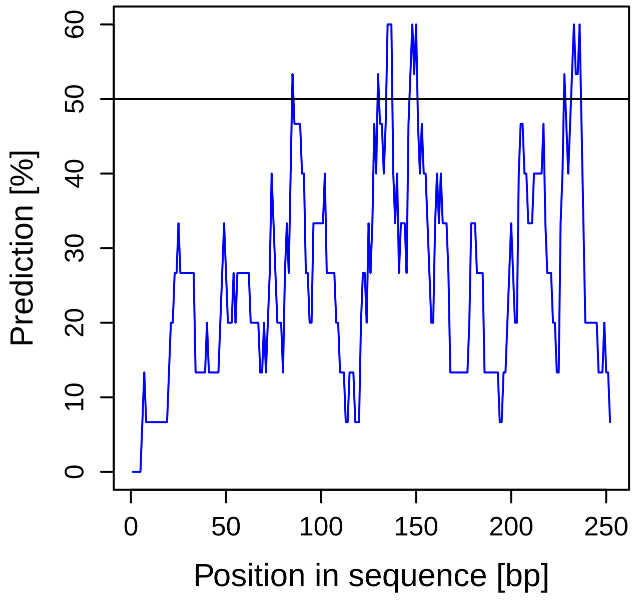

OMP_G1b: MC6_SQ01_02650

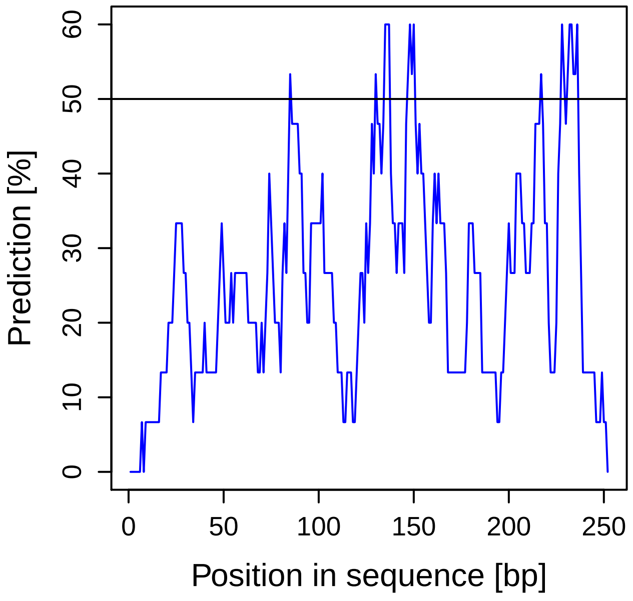

OMP_G1b: MC8_SQ02_02660

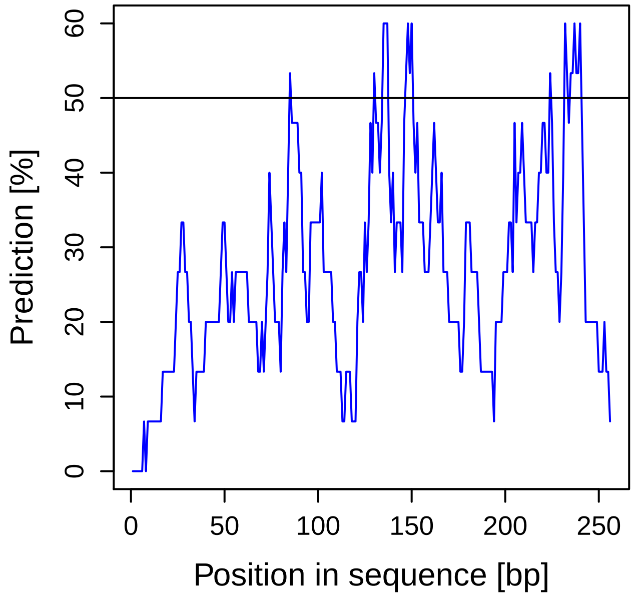

OMP_M35: MC1_SP69_05635, MC6_SQ01_03260, MC8_SQ02_03275

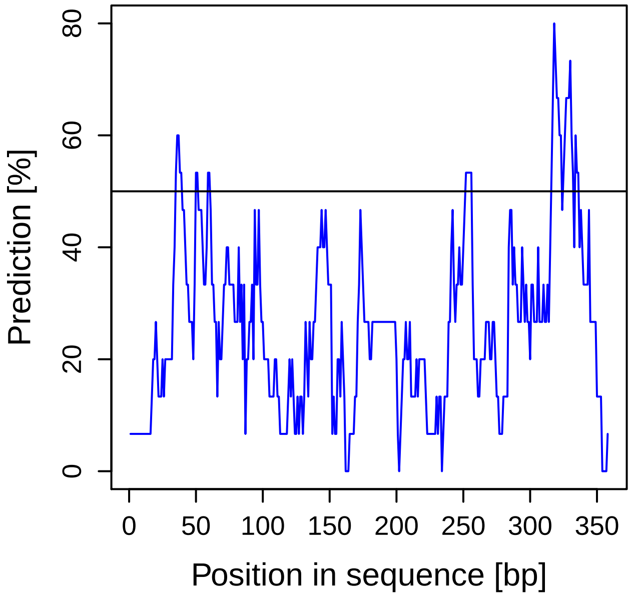

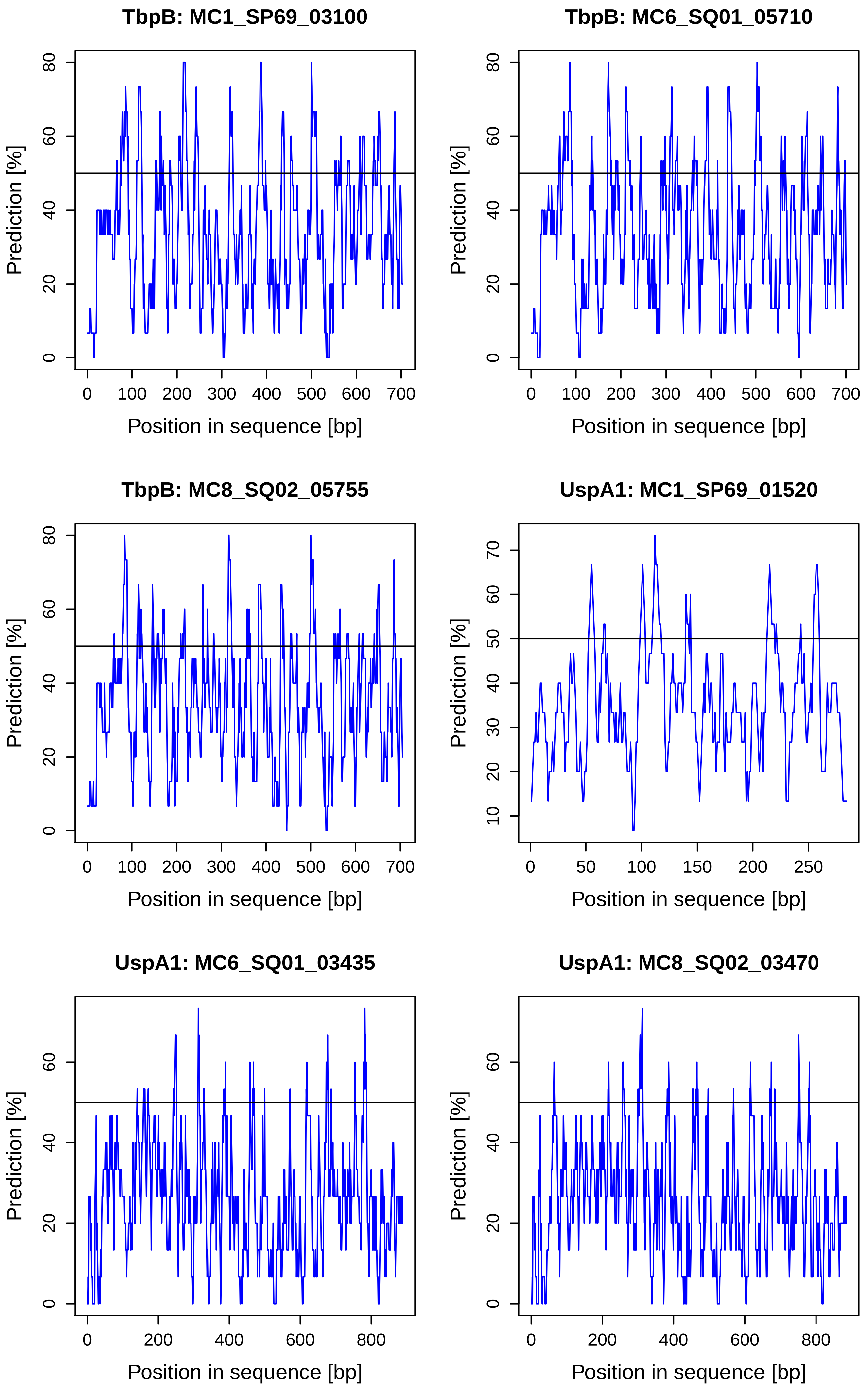
TbpB: MC1_SP69_03100
TbpB: MC6_SQ01_05710
TbpB: MC8_SQ02_05755
UspA1: MC1_SP69_01520
UspA1: MC6_SQ01_03435
UspA1: MC8_SQ02_03470
Prediction [%]
Position in sequence [bp]

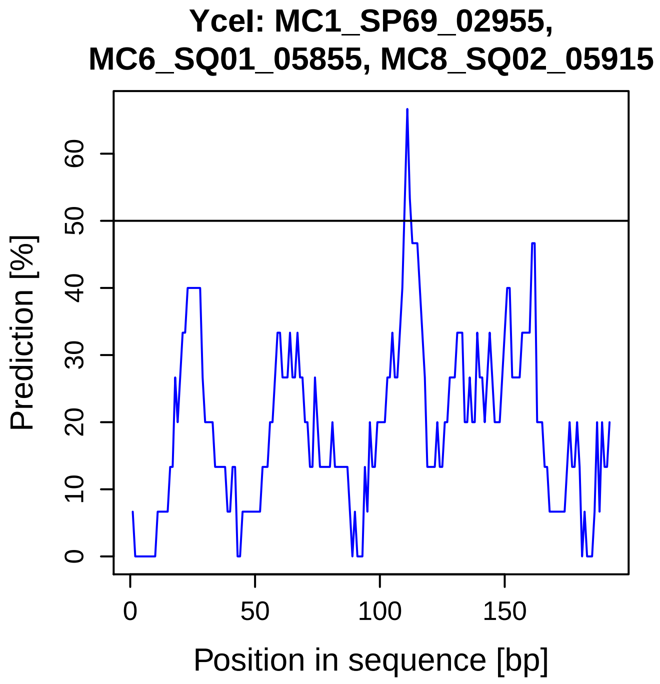

Supplementary Fig. S4. Prediction of individual sites as epitopes along sequences of proteins subjected to immunoproteomic studies from *Moraxella catarrhalis* strains MC1, MC6 and MC8. The prediction was calculated as percent of 15 methods, which recognized a given site involved in an epitope. Identical sequences were represented by one case.

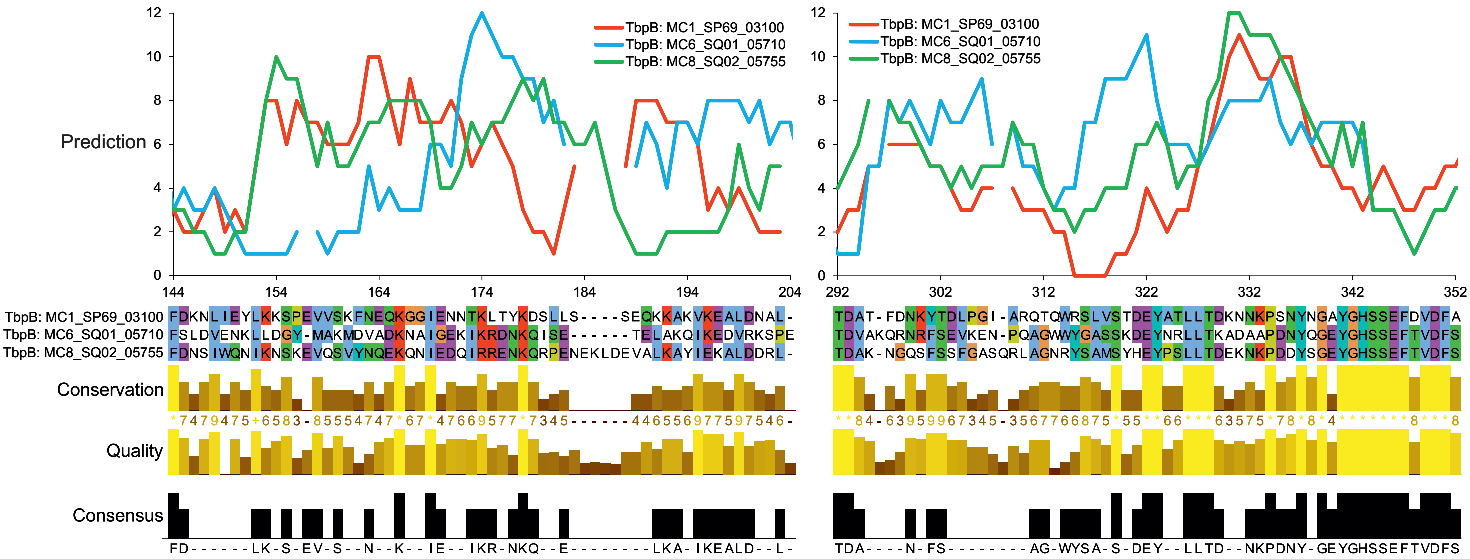

Supplementary Fig. S5. Sequence alignment of two regions in TbpB protein from *Moraxella catarrhalis* strains MC1, MC6 and MC8 and prediction of individual sites as epitopes in these sequences. The prediction was expressed as the number of 15 methods, which recognized a given site involved in an epitope. The alignment was prepared in JalView[73].
